# Temperature and Deformation-Induced Changes in the Mechanical Properties of the Amorphous Regions of Semicrystalline Polypropylene

**DOI:** 10.1021/acs.jpcb.4c07863

**Published:** 2025-01-17

**Authors:** Małgorzata Polinska, Marcin Kozanecki, Artur Rozanski

**Affiliations:** † Centre of Molecular and Macromolecular Studies, Polish Academy of Sciences, Sienkiewicza 112, Lodz 90-363, Poland; ‡ Department of Molecular Physics, Faculty of Chemistry, Lodz University of Technology, Zeromskiego 116, Lodz 90-924, Poland

## Abstract

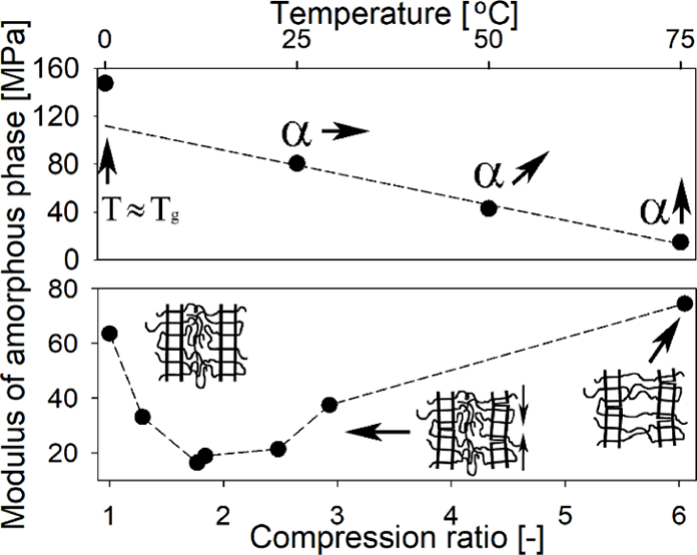

This work is focused on the impact of temperature and deformation on the mechanical properties, specifically the elastic modulus (*E*_a_) of the amorphous regions in semicrystalline polymers, using polypropylene as a case study. It has been shown that increasing temperature results in an *E*_a_ decrease due to the enhanced mobility of polymer chains, triggered by the activation of α relaxation processes within the crystalline component. Consequently, rising temperature reduces the “stiffening” effect of the crystalline regions on the interlamellar layers. Temperature decrease close to the glass transition causes a significant increase in the *E*_a_ value, reaching nearly 70 MPa. Next, the effects of crystalline/amorphous component orientation and undisturbed crystallite length on *E*_a_ were examined in materials deformed using a channel die at various compression ratios. At low compression ratios, *E*_a_ decreases nearly 4-fold, primarily due to the fragmentation of lamellar crystals in the absence of, or with relatively low, orientation of the crystalline and amorphous components. Conversely, at higher compression ratios, with minimal crystal fragmentation, increased orientation of both crystalline and amorphous regions along the deformation direction (*E*_a_ measurement direction) leads to a substantial increase in *E*_a_. Ultimately, the material with the highest used compression ratio exhibited an *E*_a_ value approximately 20% higher than that of the undeformed material.

## 1 Introduction

It is commonly accepted that the mechanical properties of semicrystalline polymers are affected by specific parameters of their microstructure. The relationship between mechanical properties and the degree of crystallinity or crystal thickness has been the topic of numerous studies.^1−5^ In addition to the degree of crystallinity and crystal thickness, one of the most extensively studied microstructural parameters is the orientation of lamellar crystals.^6−8^ The orientation observed in this class of materials results from the linear and therefore strongly anisotropic structure of the macromolecules. There are several methods for achieving significant molecular orientation in polymer materials, with the most commonly used drawing,^9^ extrusion,^10^ injection molding,^11^ and melt extrusion.^12,13^ A less common method is the channel-die compression method.^14,15^ This latter method enables the avoidance of undesirable deformation effects, such as micronecking and cavitation, which are often associated with other deformation processes, such as uniaxial stretching.^16−20^

Although the microstructure of the crystalline component is an essential factor, studies are also conducted on the influence of external factors, such as temperature and strain rate, on the mechanical response of semicrystalline polymers.^21−24^ Li et al.^25^ examined the Young’s modulus of polypropylene at different temperatures and considered the influence of the structure of the amorphous phase. Additionally, the relationship between the mechanical properties of polypropylene and the degree of crystallinity, in conjunction with temperature studies, was recently published by Li et al.^26^ It was found that at the same temperature, the value of Young’s modulus increased with the degree of crystallinity. It was also noted that mechanical characteristics, including yield strength or Young’s modulus, decrease as the temperature rises—a trend also reported for other semicrystalline polymers by Pawlak et al.,^27^ Makarewicz et al.,^20^ and Strobl et al.^28,29^ In their studies on polyethylene, they found that increasing temperature results in a lower stress that is needed to reach a given strain. Hobeika et al.^30^ showed the relationship between mechanical properties and both temperature and strain rate in a group of polyethylene materials.

The above discussion focuses on polymer characteristics concerning the crystalline structure. The microstructure and mechanical properties of the amorphous phase confined between lamellar crystals are much less-studied aspects of semicrystalline polymers, mainly due to the complex, inhomogeneous structure of these regions. Chain ends, tie molecules, entanglements, and loops reentering the crystalline lamellae are all present in the interlamellar amorphous phase. It is worth noting that only tie molecules and entanglements within the amorphous phase actively participate in stress transfer between adjacent crystals.^31^ Finally, it should be stated that the amorphous phase confined between crystals and the unconstrained amorphous phase cannot be described in the same terms regarding mechanical properties due to the aspects mentioned above, as well as the physical confinement imposed by lamellae.^32^ It is also worth mentioning that due to the irregularity resulting from the lack of long-range order, the mechanical properties of the amorphous phase are difficult to predict based on theoretical estimates or simulations.

Determining the relationship between the microstructure of material and the mechanical properties of the interlamellar amorphous phase has proven to be a challenge in many investigations. Attempts have been made to estimate the elastic modulus of the interlamellar amorphous phase (*E*_a_) using both theoretical^5^ and experimental techniques. Lame et al.^33,34^ conducted an experimental study aimed at estimating the apparent modulus of the interlamellar amorphous phase using *in situ* X-ray techniques. The results showed that the modulus of the interlamellar amorphous phase is at least an order of magnitude higher than that of the bulk amorphous phase. Unfortunately, this method has limited applicability, as it is restricted to polyethylene and requires unique equipment to collect data for estimating the modulus.

In recent work, we introduced an innovative method to measure the elastic modulus (*E*_a_) of the amorphous phase within actual polymeric (semicrystalline) materials.^35^ This technique employs a swelling agent to selectively deform only the amorphous component of the semicrystalline structure. We evaluated the local strain and stress in interlamellar regions by the observation of variations in the long period and yield stress, respectively. For high-density polyethylene (HDPE), the resulting *E*_a_ was approximately 40 MPa, markedly higher than the modulus of the bulk amorphous phase, which is close to 3 MPa.^5,36^ This increase is thought to result from an influence of the crystalline regions and the limited lateral contraction of the amorphous component due to the high aspect ratio of the lamellae. Moreover, the stiffening of the amorphous phase was influenced by tie molecules connecting lamellar crystals, as well as a higher density of entanglements within the amorphous layers.^31,37,38^ The broad applicability of our method was further confirmed through *E*_a_ measurements for other semicrystalline polymers, such as polypropylene, low-density polyethylene, or ethylene-octane copolymer.^35^

This study examines and discusses the factors behind changes in *E*_a_ values at different temperatures. Additionally, it analyzes how the crystallite size and the orientation of crystalline and amorphous components affect *E*_a_ values. These analyses were conducted on materials compressed in a channel die, a method chosen to prevent micronecking and cavitation that often arise in the uniaxial stretching of semicrystalline polymers.^16−20^ Experiments were performed with various compression ratios, and polypropylene was used as a model semicrystalline polymer for all tests.

## 2 Experimental Section

### 2.1 Materials

Commercially available polypropylene (PP) from LyondellBasell, Moplen HP456H, with a melt flow rate of 1.8 g/10 min (230 °C, 2.16 kg), was utilized in this study. The swelling agents used for this investigation were *n*-octane (for synthesis; mp −57 °C, bp 125 °C, density: 0.703 g/mL, Sigma-Aldrich) and *n*-hexane (purity: ≥99%, mp −95 °C, bp 68 °C, density: 0.655 g/mL, Sigma-Aldrich).

### 2.2 Sample Preparation

Polymer granules were compressed into 1 or 3 mm thick films at 190 °C and 50 MPa. Subsequently, the films were quenched between the metal plates. Following this, 1 mm thick samples were conditioned for 24 h at 75 °C to prevent structural alterations during mechanical and X-ray tests conducted at elevated temperatures.

**Figure 1:**
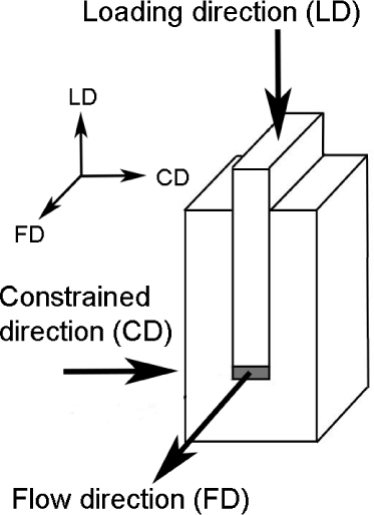
Schematic visualization of the channel die setup.

Samples with a thickness of 3 mm were compressed using the channel die setup (a schematic visualization of the device is presented in Figure 1) described in detail elsewhere.^39,40^ The compression process within the channel die was performed at 100 °C with a deformation rate of 0.001 s^–1^. To estimate the influence of the deformation on the mechanical properties of the amorphous component, samples were deformed to various strain values and then cooled to 25 °C under load. Due to the methodology of amorphous phase modulus measurement, the residual compression ratio (RCR) was utilized as an indicator of deformation degree. This parameter was determined using the following equation:
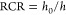
1where *h*_0_ is the initial specimen height and *h* is the height of deformed samples measured 6 months after removal from the channel die.

### 2.3 Methods

Thermal analysis was conducted using a DSC apparatus (TA Q20). Samples weighing 6–8 mg were placed into aluminum pans and tested over a temperature range of 25–190 °C, with a heating rate of 10 °C/min under a nitrogen flow. The degree of crystallinity was calculated using the following formula: *X*_c_ = Δ*H*_m_/Δ*H*_m_^0^, where Δ*H*_m_ is the measured heat of melting of the sample and Δ*H*_m_^0^ is the heat of melting of the ideal polypropylene monocrystal, assumed for this study to be equal to 209 J/g.^41^

Rectangular specimens were subjected to dynamic mechanical thermal analysis (DMTA) using a TA Q-800 apparatus (TA Instruments, New Castle, DE, USA) in the single cantilever bending mode. Measurements were conducted at a frequency of 1 Hz with a heating rate of 2 °C/min, spanning a temperature range from −50 to 100 °C, and under a constant deformation of 0.02%.

Mechanical properties were assessed using an Instron 5582 tensile testing machine equipped with a load cell range of 0–2 kN. Samples for mechanical testing were prepared in accordance with the ISO 527–2 standard, employing dog-bone-shaped samples with a 25 mm gauge length, 5 mm width, and 1 mm thickness. They were subjected to stretching at a constant rate of 3.3 × 10^–3^ s^–1^ (corresponding to a crosshead speed of 5 mm/min). Tensile measurements were conducted within an environmental chamber at four different temperatures (0, 25, 50, and 75 °C).

The lamellar structure of samples was probed with two-dimensional small-angle X-ray scattering (2-D SAXS). The Kiessig-type camera with a sample detector distance of 1.2 m was coupled to an X-ray Cu–Kα low divergence microsource, operating at 50 kV and 1 mA (sealed-tube microsource integrated with multilayer collimation optics, producing a highly collimated beam with a divergence of 0.8 × 0.8 mrad^2^;^2^ GeniX Cu-LD by Xenocs, France). The collimation optics were combined with two additional hybrid scatterless slit systems (Xenocs) placed between the multilayer optics and the sample stage, forming the beam of square cross-section. The two slit assemblies were separated by 1200 mm. The radiation scattered by the sample was recorded with the Pilatus 100 K solid-state area detector with a resolution of 172 × 172 μm^2^ (Dectris, Switzerland). The long period was determined from one-dimensional sections of the 2-D pattern. Background and Lorentz corrections were applied to the curves. Then, the long period was calculated from the position of the maximum of the corrected curves using Bragg’s law. In the case of oriented materials (compressed in the channel die), the X-ray beam penetrated the sample along the loading direction, and long period measurements were conducted in the flow direction (compare to Figure 1).

The crystalline structure of the materials was analyzed by using wide-angle X-ray scattering (WAXS) with a computer-controlled goniometer connected to a sealed-tube Cu–Kα radiation source (Philips), operating at 50 kV and 30 mA. A standard Ni filter was used alongside electronic filtering to isolate the Cu–Kα line, and data collection occurred in reflection mode.

The characteristic signals from the monoclinic form of polypropylene were examined from the (110), (040), and (130) crystallographic planes to determine the interplanar distance and crystallite size perpendicular to these planes, using the Scherrer formula:
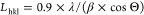
2where *L*_hkl_ is a crystallite length in the direction perpendicular to the (hkl) plane, λ is the X-ray wavelength, β is the half-width of a diffraction peak, and Θ is the Bragg’s diffraction angle. The half-widths of the analyzed peaks were determined by deconvoluting the X-ray profiles using WAXSFit software.^42,43^ The half-widths of the diffraction peaks were corrected for apparatus broadening.

**Figure 2:**
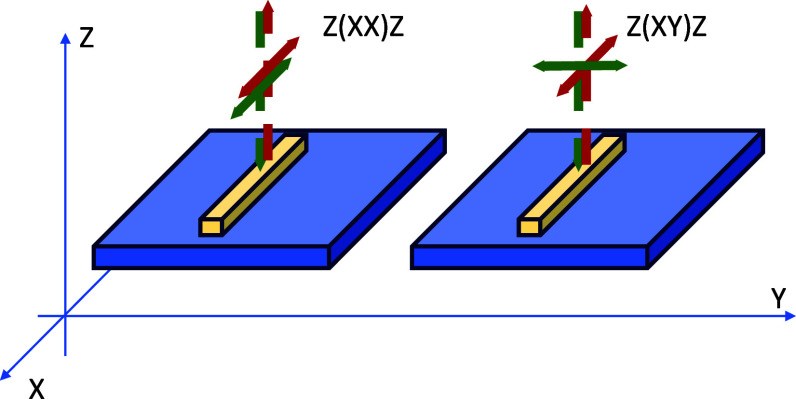
Measurement configurations used for polarized Raman scattering experiments.

Polarized Raman microspectroscopy was used to determine the orientation of polymer segments in samples prepared by compression within the channel die based on the ratio of the intensities of the bands assigned to CH_2_ and C–C groups for parallel (Z(XX)Z) and perpendicular (Z(XY)Z) configurations (see Figure 2). Polymer chain orientation in both amorphous and crystalline phases was analyzed based on the multicomponent Raman band located between 800 and 850 cm^–1^.^44^

Raman spectrometer Jobin Yvon T64000 equipped with an Olympus BX-40 confocal microscope was used. All measurements were performed with an excitation wavelength of 514.5 nm and a laser beam power of 1.5 mW at the sample surface. Spectra were collected in the wavenumber range of 750–875 cm^–1^ with a spectral resolution of c.a. 0.5 cm^–1^. The samples were placed on the microscope stage and measured two times for 30 s per point. The measurements were done for two angles between the polarization of incident light and the expected direction of chain orientation (flow direction), labeled as 0° (for parallel configuration) and 90° (for the perpendicular one).

PeakFit software was used to subtract the baseline from the raw spectra and then perform deconvolution. Two peaks at 809 and 842 cm^–1^ characteristic for the crystalline phase and the line at 830 cm^–1^ assigned to the amorphous phase were separated according to the literature.^44^ The peak positions were locked, and the integral intensity of each band was determined. The exemplary results of the deconvolution process performed for samples with RCR 1 and 6.05 are presented in Figures S1 and S2, respectively.

Obtained integral intensities of chosen Raman peaks were used to calculate the depolarization ratio for the amorphous component, as described below:
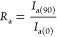
3where *R*_a_ is the depolarization ratio for the amorphous phase; *I*_a(90)_ is the intensity of the 830 cm^–1^ band at an angle of 90° between the polarization of the incident light and the expected chain orientation; and *I*_a(0)_ is the intensity of the 830 cm^–1^ band at an angle of 0° between the polarization of the incident light and the expected chain orientation.

To estimate the depolarization ratio for the crystalline component, the following equation was used:
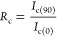
4where *I*_c(90)_ and *I*_c(0)_ are the intensities of the crystalline bands, represented by the following equation:
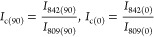
5

The subscript numbers, i.e., 809 and 842, indicate the position of the bands characteristic for the crystalline phase, and the numbers in parentheses, i.e., (0) and (90), indicate the angle between the polarization of the incident light and the expected direction of chain orientation.

## 3 Results

### 3.1 Determination of the Elastic Modulus of the Interlamellar Amorphous Phase

The method for determining the elastic modulus of the interlamellar amorphous phase, which involves changing of the interlamellar distance through selective swelling of the interlamellar amorphous phase, has already been thoroughly described in previous works.^35,45^ Briefly, to determine the value of the elastic modulus of the interlamellar amorphous phase, eq 6 is utilized:
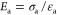
6where *E*_a_ is the elastic modulus of the interlamellar amorphous phase, σ_a_ and ε_a_ denote the swelling-induced local stress and local strain of the interlamellar amorphous layers, respectively. Therefore, it is first necessary to obtain swelling-induced mechanical parameters, such as local stress (σ_a_) and local strain (ε_a_) for the interlamellar amorphous regions.

#### 3.1.1 Local Strain of the Interlamellar Amorphous Phase—ε_a_

As previously stated, the method involves the introduction of a carefully selected low molecular weight substance that causes selective swelling by specifically penetrating only the amorphous component. This process does not affect the crystalline phase, as has been demonstrated with hexane and other swelling agents.^46,47^ Experiments demonstrating the nonpenetration of *n*-octane into the crystalline regions, as evidenced by the absence of significant changes in interplanar distances and the undisturbed length of crystals, are presented in Figure S3 and Table S1. The analysis confirms that octane neither infiltrates the crystalline component nor causes the dissolution of polypropylene crystals. Hence, the observed change in the long period (LP, indicative of the mean thickness of amorphous and crystalline layers) between the reference and swollen samples can be attributed to a change in the distance between adjacent lamellar crystals (eq 7).
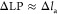
7where ΔLP is a change of long period and Δ*l*_a_ is the swelling-induced change of thickness of the amorphous layers.

Taking the above into account, the value of the ε_a_ parameter can be determined from a simple relationship between the swelling-induced change in thickness of the amorphous layers (corresponding to the change in long period, ΔLP) and their initial thickness (eq 8):
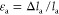
8where *l*_a_ is the initial thickness of the amorphous layers.

#### 3.1.2 Local Stress of the Interlamellar Amorphous Phase—σ_a_

To establish the local stress in the interlamellar amorphous phase, changes in the mechanical properties between swollen and “dry” samples were analyzed. A measurable decrease in yield stress was observed in the swollen materials.^48−50^ This effect was attributed to a change in the stress state of the molecules connecting adjacent crystals (tie molecules) during the swelling process. Consequently, the lamellar crystals were in a predeformation state, requiring lower stress to initiate their plastic deformation. The macroscopic manifestation of this phenomenon was a lower yield stress. Since the crystalline and amorphous components are physically connected, the value of stress within the amorphous regions must be analogous. This leads to the following equation (eq 9):
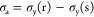
9where σ_a_ is the swelling-induced local stress of the interlamellar amorphous phase, σ_y_(r) is the yield stress of the reference sample (before the modification process), and σ_y_(s) is the yield stress of the swollen sample.

The swelling-induced local stress of the interlamellar amorphous phase of compressed materials was determined by using an alternative method. In this approach, the swollen sample, removed from the hexane bath, was clamped in a tensile testing machine, and the stress buildup in the sample was recorded as a function of the desorption time of the swelling agent. During desorption, contraction of the amorphous regions back to their initial state was restricted. This lack of free contraction during the evaporation of the swelling agent generated stress within the amorphous phase, which was transferred through the crystalline component and further along the sample. The macroscopic manifestation of this phenomenon was the stress measured by the crosshead of the tensile device. Naturally, the value of stress determined in this experiment corresponds to the local stress generated in the interlamellar amorphous regions as a result of the swelling process. As demonstrated in our previous work,^35^ the local stress values determined from changes in yield stress and the stress “buildup” experiment during swelling agent desorption, for the same material, were identical.

### 3.2 The Structure of PP Samples

In this study, two separate sets of polypropylene (PP) samples were used. The structure of the first set was uniform/isotropic. These samples were used to examine the influence of temperature (ranging from 0 to 75°C) on the mechanical properties of amorphous regions. They were designed as PP_(0)_, PP_(25)_, PP_(50)_, and PP_(75)_, where the subscript number represents the temperature at which measurements were taken. Due to the elevated temperature, *n*-octane was used as a swelling agent, as its evaporation at the selected temperatures (particularly 50 and 75 °C) would be limited.

The second set of samples, compressed in a channel die, was used to examine the influence of the deformation process on the interlamellar amorphous phase modulus. These samples were designed as PP_(1)_, PP_(1.29)_, PP_(1.77)_, PP_(1.84)_, PP_(2.48)_, PP_(2.93)_, and PP_(6.05)_. The subscript number indicates the value of the residual compression ratio (RCR), determined from the reduction of the specimen weight (along the loading direction) according to eq 1. A RCR value of 1 corresponded to an undeformed sample. A representative compression curve with marked RCR values analyzed in this work is shown in Figure S4. The modulus measurements for these samples were carried out along the flow direction at room temperature (RT = 25 °C), using *n*-hexane as a swelling agent.

Samples from both sets were characterized in terms of microstructure using the X-ray scattering technique (SAXS) combined with calorimetry (DSC) prior to modification. The parameters obtained from these measurements are shown in Table 1.

**Table 1:** Selected Structural Parameters of Analyzed Polypropylenes before Modification

Sample name	Crystalline mass fraction, *X*_c_ [%]^a^	Crystalline volume fraction, *X*_v_ [%]^b^	Long period (LP) of an unswollen sample [nm]^c^	Thickness of crystalline layers, *l*_c_ [nm]^d^	Thickness of amorphous layers, *l*_a_ [nm]^d^
PP_(0)_	43.6	39.7	14.4 ± 0.3	5.7 ± 0.2	8.7 ± 0.2
PP_(25)_			14.6 ± 0.4	5.8 ± 0.2	8.8 ± 0.2
PP_(50)_			14.1 ± 0.3	5.6 ± 0.2	8.5 ± 0.2
PP_(75)_			14.0 ± 0.2	5.6 ± 0.1	8.4 ± 0.1
PP_(1)_	38.9	35.2	14.4 ± 0.0	5.1 ± 0.0	9.3 ± 0.0
PP_(1.29)_	38.7	35.1	16.2 ± 0.2	5.7 ± 0.1	10.5 ± 0.1
PP_(1.77)_	40.0	36.3	14.3 ± 0.1	5.2 ± 0.1	9.1 ± 0.1
PP_(1.84)_	41.3	37.6	14.7 ± 0.4	5.5 ± 0.2	9.2 ± 0.2
PP_(2.48)_	40.5	36.8	14.5 ± 0.6	5.3 ± 0.2	9.2 ± 0.3
PP_(2.93)_	41.1	37.3	14.7 ± 1.0	5.5 ± 0.4	9.2 ± 0.7
PP_(6.05)_	40.9	37.2	14.0 ± 0.2	5.2 ± 0.1	8.8 ± 0.1

a from DSC.

b by transforming the crystalline mass fraction with the use of densities of crystalline and amorphous components (*d*_c_ = 0.949 g/cm^3^, *d*_a_ = 0.854 g/cm^3^).^51^

c from SAXS.

d estimated from crystalline volume fraction and LP.

The structural parameters of the isotropic samples were typical for slowly solidified and annealed (at 75 °C) polypropylene. The parameters of the crystalline and amorphous regions as a function of temperature did not change significantly. Only in the case of the amorphous layers, a small decrease in their thickness was observed at elevated temperatures, accompanied by a decrease in the LP value, probably induced by the higher mobility of the chain network and thermal “collapse” of these regions.

Table 1 also presents the degrees of crystallinity for both undeformed and compressed materials. The degree of crystallinity for undeformed polypropylene (PP_(1)_) was lower than that in the sample PP_(25)_. This effect was caused by the different thermal histories of the two sets of samples with an additional annealing stage at 75 °C in the case of the PP_(25)_ material. In the analyzed range of RCR values, a gradual increase in the degree of crystallinity was observed. However, in the PP_(6.05)_ sample, the *X*_c_ value was only 5% higher than that of the undeformed sample. This effect was likely induced by the annealing process applied to the compressed samples within a channel die during deformation at 100 °C, followed by slow postdeformation cooling to room temperature.

**Figure 3:**
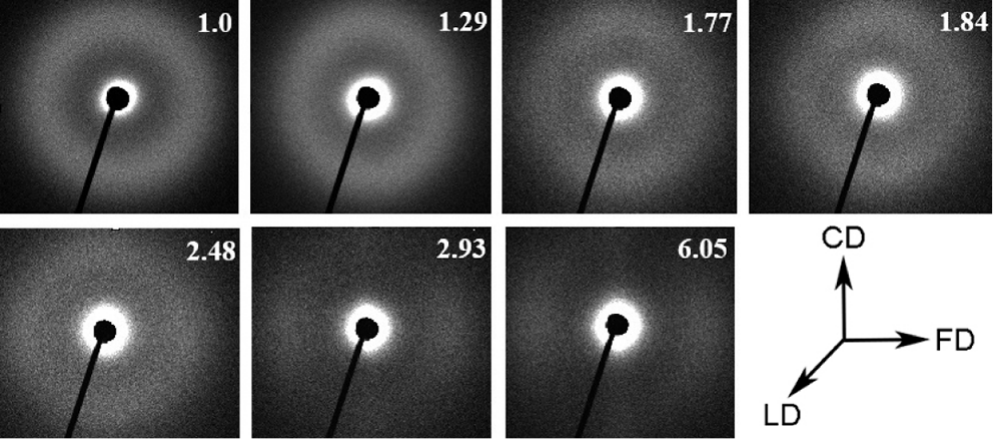
Small-angle X-ray scattering (SAXS) patterns for samples with increasing values of the residual compression ratio. X-ray illumination along the loading direction (LD). Flow direction (FD): horizontally; constrained direction (CD): vertically. The numbers correspond to the RCR values.

Additionally, Table 1 presents the changes in the value of the long period calculated from the one-dimensional profiles along the flow direction (FD) of the two-dimensional SAXS patterns shown in Figure 3. The LP was identical in both PP_(1)_ and PP_(25)_, however, the crystal thickness was higher in the latter sample. This effect was caused by different thermal histories, as mentioned in the previous paragraph. With the increase of RCR, the LP initially significantly increased and then decreased below the value observed for undeformed material. The observed increase in the LP value for the PP_(1.29)_ sample could be induced by the accumulation of deformation mainly within the amorphous regions. Therefore, the *l*_a_ and *l*_c_ values for the PP_(1.29)_ sample could be slightly underestimated and overestimated, respectively. A similar trend in LP changes along the flow direction was observed in other studies.^52^

In order to track the evolution of the polypropylene microstructure with increasing RCR, two parameters were analyzed: the orientation of the crystalline and amorphous components and the undisturbed crystallite length.

Deformation-induced changes in the crystalline/amorphous component’s orientation were studied using X-ray techniques and Raman spectroscopy. Figure 3 illustrates the two-dimensional small-angle X-ray scattering (SAXS) patterns, collected in the flow direction (FD)-constrained direction (CD) plane, with the samples illuminated along the loading direction (LD). Previous studies have examined the microstructural evolution of compressed polypropylene in detail.^14,53^ Briefly summarizing, as the RCR increased from 1 to 1.29, the SAXS pattern, initially circular, transitioned into an elliptical shape. This transformation indicates that the deformation of polypropylene induces microstructural changes, notably an increase in interlamellar distances for lamellae oriented perpendicular to the FD, as reflected by the rise in LP values along the FD (see Table 1). These changes are accompanied by a crystallographic slip and lamellar sliding. At higher RCR values, the modifications in the SAXS patterns suggested the formation of a new microstructure, characterized by the development of a new long period along the FD, gradually replacing the initial structure. In the sample compressed to an RCR of 6.05, a distinct two-point scattering pattern emerged, indicating that the crystallites were mainly oriented perpendicular to the FD. This also suggested that lamellae aligned with the CD either reoriented or underwent significant structural changes. Notably, this new long period was lower than that observed in the undeformed sample (Table 1).

**Figure 4:**
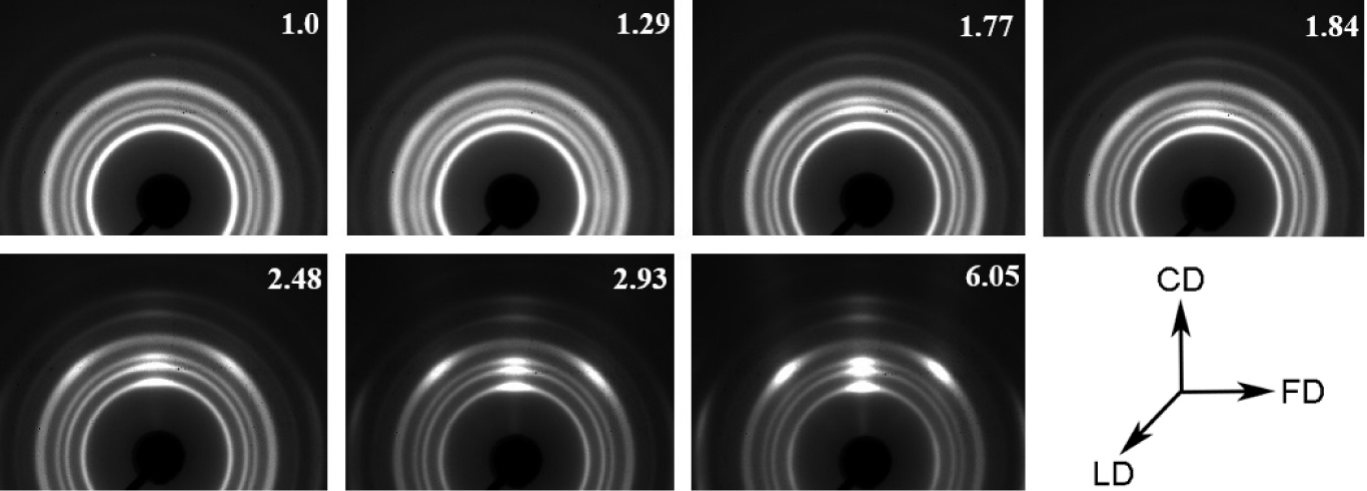
Wide-angle X-ray scattering (WAXS) patterns for samples with increasing values of the residual compression ratio. X-ray illumination along the loading direction (LD). Flow direction (FD): horizontally; constrained direction (CD): vertically. The numbers correspond to the RCR values.

The evolution of crystalline microstructure with rising RCR values, as observed using the WAXS technique (Figure 4), is consistent with the trends shown by SAXS. As the RCR increases, there is a progressive alignment of the (110), (040), and (130) planes within the polar region of the WAXS patterns. When considering the orientation of these planes relative to the lamellar crystal plane, it becomes evident that, in the sample subjected to the highest compression (RCR = 6.05), most of the crystals have their normals aligned either parallel or at an insignificant angle to the FD.

In the case of polypropylene, the phenomenon of lamella cross-hatching is observed. For nonoriented or weakly oriented materials, signals from cross-hatched lamellae will not be visible in WAXS patterns. During compression in the channel die, fragmented crystals (as discussed later in the article), regardless of whether they originate from mother or daughter (cross-hatched) lamellae, undergo gradual orientation in the flow direction (FD). Therefore, the orientation of crystals in a single preferred direction is observed in the WAXS patterns.

**Figure 5:**
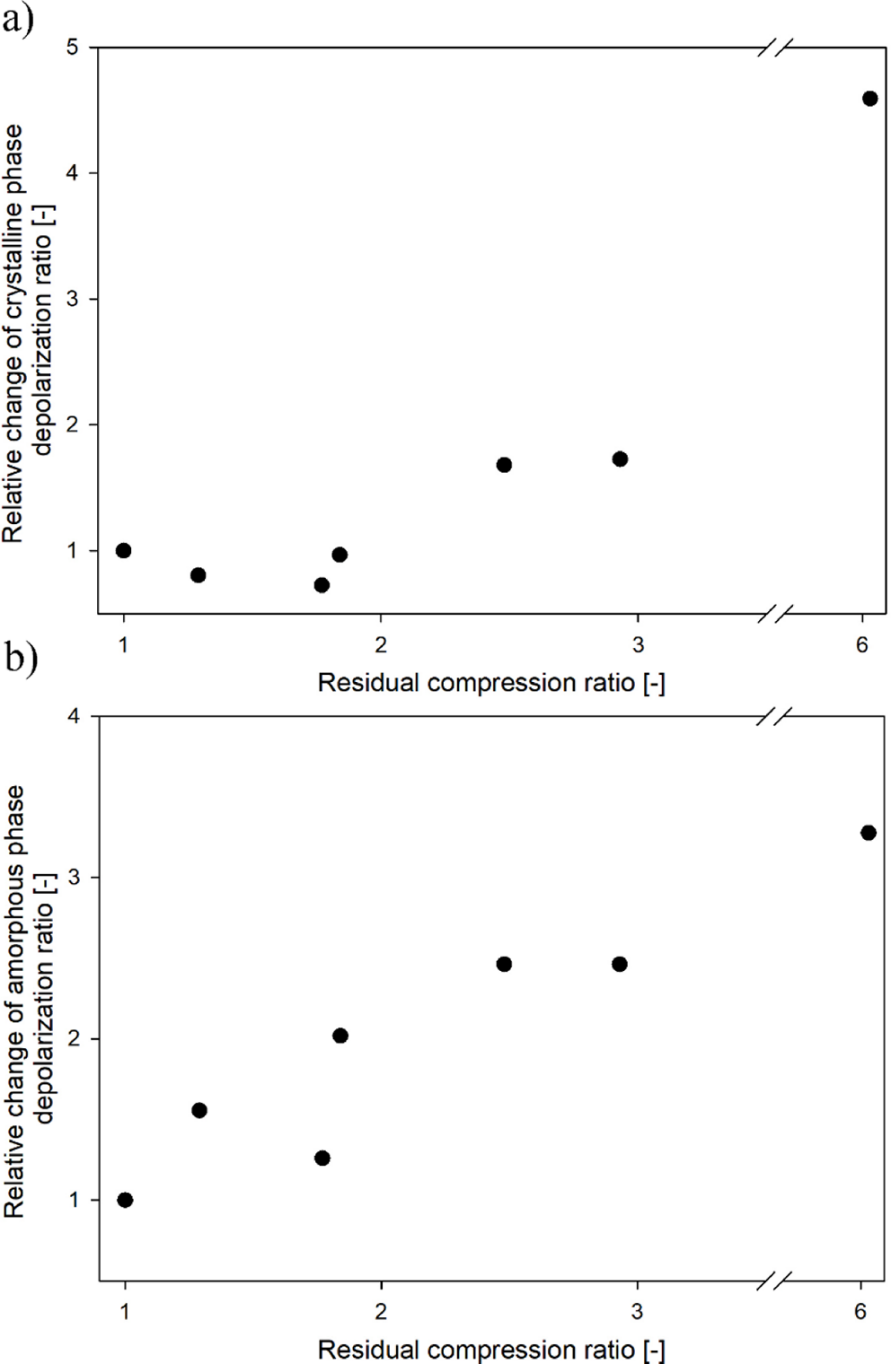
Depolarization ratio of the crystalline (a) and amorphous (b) phases, calculated from Raman spectra, as a function of the residual compression ratio.

Additionally, to quantitatively characterize the changes in the orientation of crystalline and amorphous components in compressed samples across the analyzed range of RCR values, Raman spectroscopy was used. The results of the depolarization ratio calculations for the crystalline and amorphous components, following the methodology outlined in eqs 3 and 4, are shown in Figure 5a,b, respectively.

Due to the difference in mechanical properties between the crystalline and amorphous components, initially, up to an RCR value of 1.84, the deformation of the material accumulates in the interlamellar regions. This is accompanied by an increase in the depolarization ratio determined for the amorphous component. At this stage of deformation, the crystalline phase remains unoriented or slightly oriented outside the FD (hence, the lower depolarization ratio compared to the undeformed sample). This effect was previously observed in polypropylene and was caused by the presence of cross-hatched lamellae.^54^ As the RCR increases, the deformation of the amorphous layers becomes almost exhausted, after which the crystalline phase begins to deform, resulting in a high orientation. Finally, at an RCR of 6.05, the chains in both the crystalline and amorphous phases become highly oriented in the FD. At this stage of deformation, the orientation of polypropylene chains in the FD in the crystalline and amorphous components is ≈5 and ≈3 times higher, respectively, than in the CD. It is worth noting that the gradual stretching and orientation of the molecular lattice in the amorphous regions will introduce new spatial constraints on this component, which is expected to increase the *E*_a_ value.

In our recent work,^35^ we postulated that the increase in the elastic modulus of the amorphous component confined between lamellae, compared to bulk amorphous material, is induced, among other factors, by the limited lateral contraction in the amorphous phase, due to the lateral extent of lamellar crystals with a very high aspect ratio. Simultaneously, during polymer deformation, the fragmentation of lamellar crystals is observed, which should favor the lateral contraction of chains within the amorphous regions, resulting in the decrease of *E*_a_ value. It is difficult to precisely track changes in the size of lamellae (as a whole) during the polymer deformation process. However, the Scherrer method (eq 2) can be used to measure the undisturbed dimensions of crystallites (in the lamellae lateral directions), as the fragmentation process at the lamella level also affects the undisturbed crystallite length.

The peaks of the (110) and (040) crystallographic planes of the polypropylene monoclinic form were specifically chosen for consideration. This selection of crystallographic planes was deliberate. As presented above, during the compression of PP in a channel die, a single-component crystalline texture is formed. In this texture, most of the crystals are oriented with the normal of the (hk0) planes in the CD–LD plane. Therefore, analyzing the peaks of the (110) and (040) planes along the CD would provide information about changes in the crystal dimensions for lamellae oriented with their normals along the FD (the direction of *E*_a_ measurements).

The WAXSFit software was used to deconvolute WAXS profiles collected along the CD (as illustrated in Figure S5) and to evaluate the half-width of specific peaks. This analysis included both crystalline and amorphous phases. Figure S6 presents exemplary deconvolution results for selected materials. The undisturbed crystallite lengths were then estimated according to eq 2. For undeformed PP, the crystallite lengths in the direction perpendicular to the (110) and (040) planes were found to be 18.4 and 20.0 nm, respectively.

**Figure 6:**
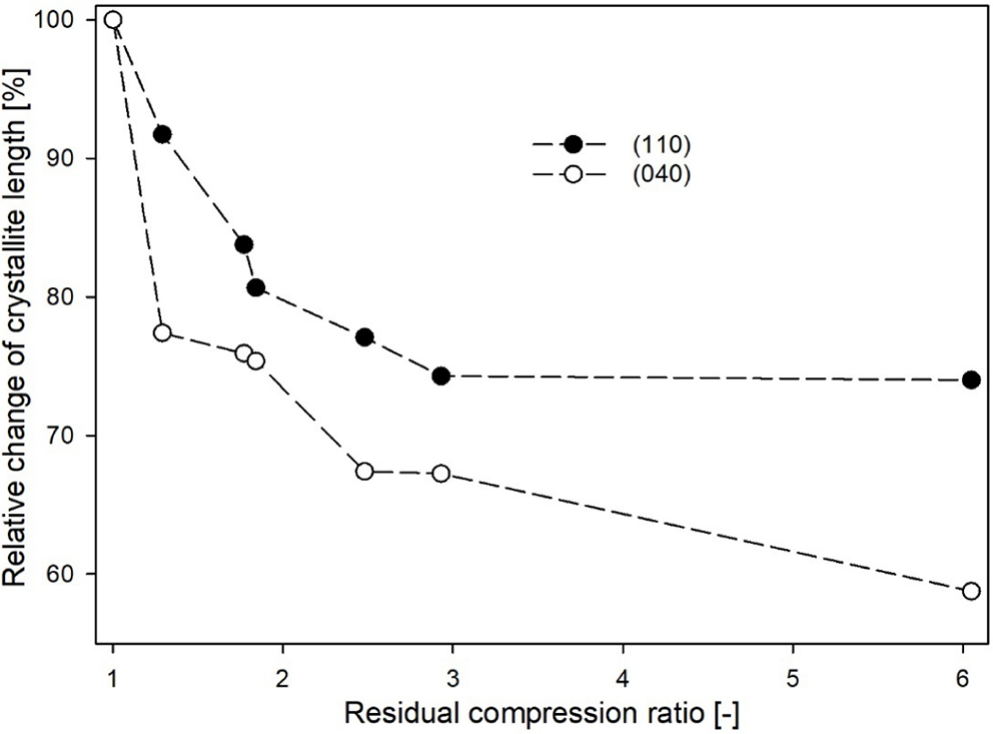
Relative change of undisturbed crystallite length in the normal direction to the (110) and (040) planes along the CD as a function of the residual compression ratio. The dashed lines are drawn only for eyes guidance.

Figure 6 shows the relative variations in undisturbed crystallite length measured perpendicular to the examined crystallographic planes as a function of the RCR. The results indicate that the crystallite length responded similarly to RCR changes across the analyzed planes. However, the reduction in crystal dimensions was more pronounced along the normal to the (040) plane. The changes in undisturbed crystallite length are most dynamic up to a RCR value of 2.48, suggesting lamellae kinking and fragmentation. For materials with higher RCR, no further significant changes in undisturbed crystallite length are observed.

### 3.3 Elastic Modulus of the Interlamellar Amorphous Phase—Influence of Temperature

The *E*_a_ of polypropylene at four temperatures (0, 25, 50, and 75 °C) was determined. The chosen temperature range was intentional as the glass transition typically occurs in PP in the range of −20 to 0 °C^55,56^ (see also Figure S7), and at elevated temperatures, the α relaxation process in PP is additionally observed. The α relaxation is a complex process involving simultaneous relaxation in the crystalline phase and at the crystal–amorphous interphase.^57^ Recently, analyzing polyethylene with different thicknesses of crystals, we demonstrated that the mechanical properties of the amorphous regions are correlated with the α relaxation process.^45^ When the material is below the temperature of α relaxation at a given temperature, the stiffening influence of the crystals on the amorphous component becomes more pronounced. As shown in Figure S7, the α relaxation process in the analyzed polypropylene is activated above 50 °C, with an apparent maximum at around 90 °C. Therefore, the proposed temperature range allows for the characterization of the mechanical properties of polypropylene’s amorphous regions, both near the *T*_g_ and at various “intensities” of the α relaxation process.

**Figure 7:**
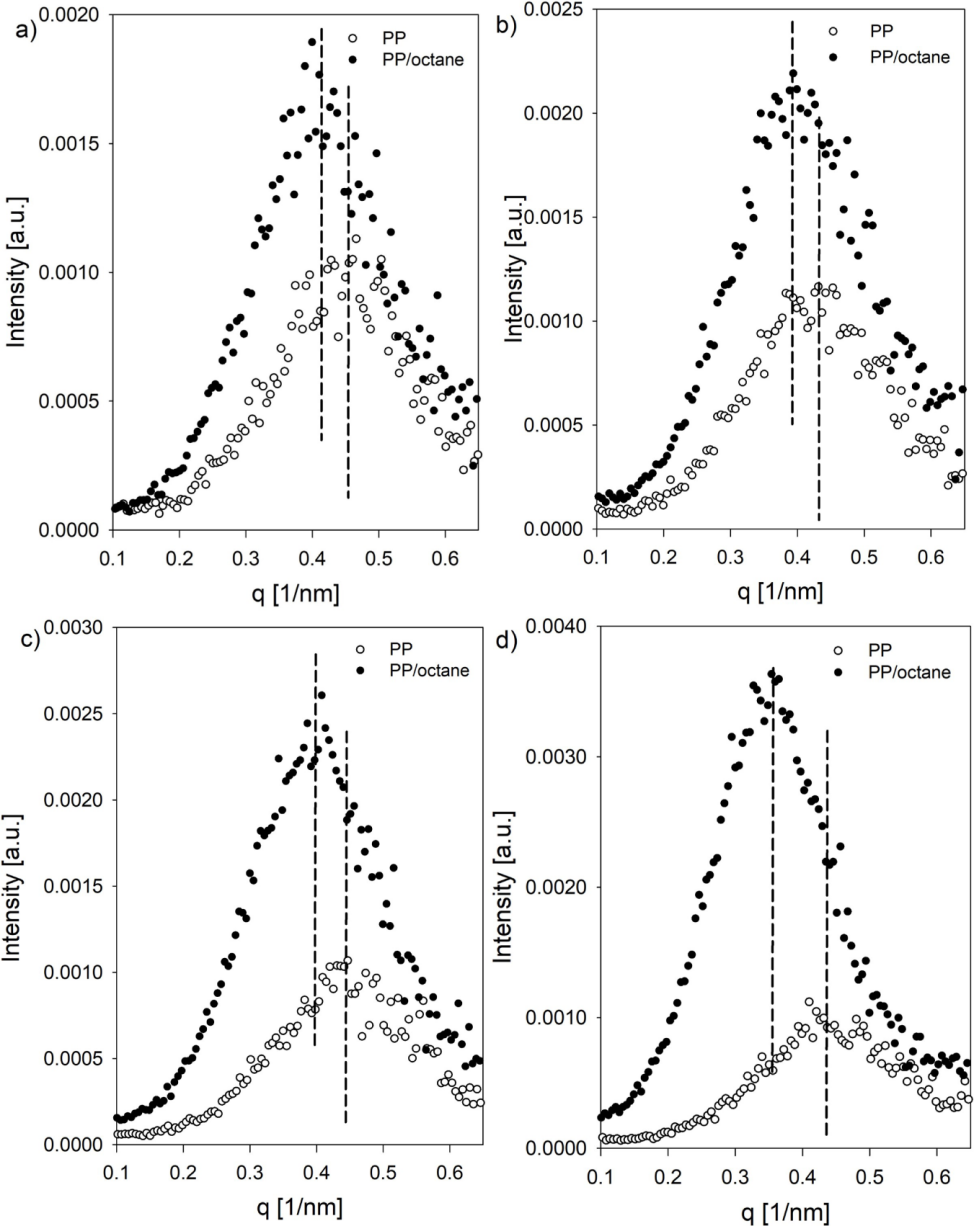
SAXS profiles for reference (PP) and swollen (PP/octane) samples as a function of temperature: a) 0 °C, b) 25 °C, c) 50 °C, and d) 75 °C.

Figure 7 presents SAXS profiles recorded for reference and swollen samples as a function of temperature. In all analyzed materials, the introduction of octane caused a shift of the maximum of the *q* profile toward lower values, indicating an increase in interlamellar distances. Notably, the swelling-induced shift increased noticeably with rising temperature. By examining the *q* profile maxima of both neat and octane-swollen systems, we determined the changes in the thickness of the interlamellar layers (see Table 2). Subsequently, using the data on the initial thickness of the amorphous layers of reference samples (Table 1), we estimated the local strains of the amorphous layers (eq 8 and Table 2). The data presented in Table 2 support the earlier observation that the susceptibility of interlamellar regions to swelling increases with temperature. Consequently, the measured value of the local strain increases with temperature.

**Table 2:** Structural and Mechanical Parameters for Octane-Swollen Systems

Sample	Change of Long Period (LP) [nm]^a^	Local strain of amorphous phase [-]^b^	Yield stress of reference PP [MPa]^c^	Yield stress of swollen PP [MPa]^c^	Local stress of amorphous phase [MPa]^d^	Elastic modulus [MPa]^e^
PP_(0)_	0.8	0.092	39.7 ± 2.2	26.1 ± 0.4	13.6 ± 0.5	147.8 ± 5.4
PP_(25)_	1.1	0.125	31.1 ± 0.7	21.0 ± 0.4	10.1 ± 0.2	80.8 ± 1.6
PP_(50)_	1.7	0.200	20.9 ± 1.5	12.3 ± 0.4	8.6 ± 0.6	43.0 ± 3
PP_(75)_	3.9	0.464	14.9 ± 0.7	8.0 ± 0.5	6.9 ± 0.1	14.9 ± 0.2

a from SAXS.

b in accordance with eqs 7 and 8.

c from mechanical measurements.

d in accordance with eq 9.

e in accordance with eq 6.

**Figure 8:**
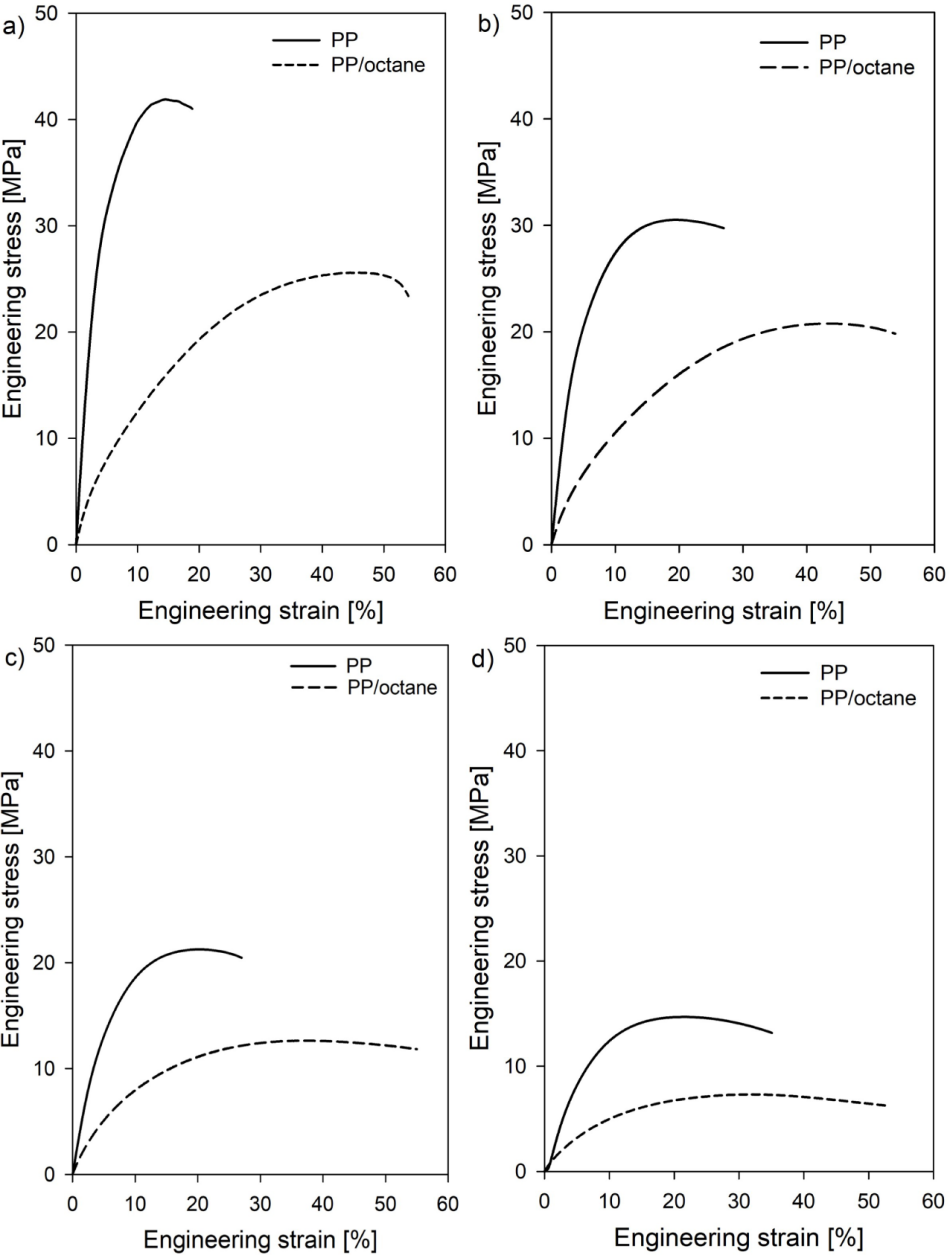
Mechanical curves for reference (PP) and swollen (PP/octane) samples as a function of temperature: a) 0 °C, b) 25 °C, c) 50 °C, and d) 75 °C.

Figure 8 presents representative mechanical curves collected for reference polypropylene and *n*-octane-swollen systems. Changes in deformation temperature noticeably affected the mechanical response of the “dry” polypropylene, with a systematic decrease in yield stress observed as the temperature increased. A similar effect of deformation temperature on the mechanical properties of semicrystalline polymers has been reported by others.^27,58,59^ The presence of the swelling agent caused a reduction in yield stress in the studied systems, reflecting the level of prestress generated within the crystals and, as discussed earlier, the swelling-induced local stress in the interlamellar layers. Notably, the swelling-induced decrease in yield stress became less pronounced with increasing temperature. The values of σ_y_(d), σ_y_(s), and the swelling-induced local stress in the amorphous layers (σ_a_) for polypropylene materials as a function of temperature are presented in Table 2.

**Figure 9:**
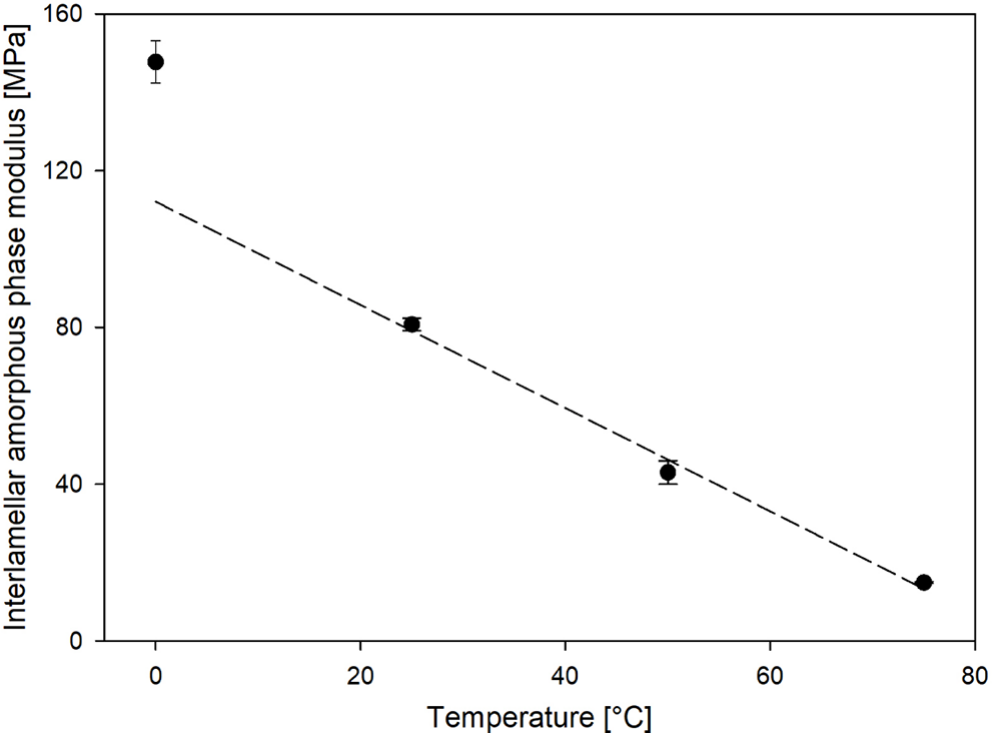
Interlamellar amorphous phase modulus of PP in the course of temperature. The dashed line is drawn only for eyes guidance.

The values of local strain and stress (Table 2) were utilized to calculate the elastic modulus of the amorphous regions following the methodology outlined in eq 6 (Table 2). Furthermore, Figure 9 illustrates the variation of *E*_a_ with temperature. As seen, the mechanical properties of the amorphous regions varied significantly with temperature. At room temperature, the *E*_a_ value was higher than that reported in our previous study.^35^ This effect was caused by the higher thickness of the crystal in the annealed sample analyzed in the present work and the direct influence of crystal thickness on the *E*_a_ value, which we demonstrated in recent studies.^45^

As the temperature for measuring *E*_a_ increased, a gradual decrease in the stiffening of the amorphous regions was observed. In the temperature range of 25–75 °C, practically, a linear decrease in the *E*_a_ value with temperature was observed. This effect was caused by a gradual increase in the mobility of macromolecular fragments in the crystalline regions resulting from the α relaxation process at elevated temperatures. Consequently, this led to a gradual reduction of the “stiffening” effect of the lamellar crystals on the amorphous regions. Moreover, lowering the temperature to 0 °C caused a significant increase in the *E*_a_ value, reaching nearly 70 MPa. Simultaneously, a deviation from the linear trend of *E*_a_ change with temperature, observed at higher temperatures, was noted. This effect is due to the proximity of the glass transition temperature of the disordered regions, as shown in Figure S7.

### 3.4 Elastic Modulus of the Interlamellar Amorphous Phase—Influence of Deformation Process

The local strain in the amorphous phase (ε_a_), caused by swelling, was calculated as previously outlined by evaluating the changes in the long period value along the FD. Figure S8 displays SAXS profiles for both undeformed and compressed materials, taken before and after swelling. Introducing hexane led to a shift in the *q* profile maxima toward lower values, similar to observations from the temperature experiments. Notably, the shift in the *q* profile due to swelling for the undeformed sample resembled what was seen in the PP_(25)_ sample (referenced in Subsection 3.3). However, for samples compressed within an RCR range of 1.29–2.48, the shift was more significant, decreasing sharply for more compressed samples, especially for the sample with an RCR of 6.05.

By analyzing the positions of the *q* profiles maxima for both pure and hexane-swollen systems, the changes in the long period and, consequently, the thickness of the interlamellar layers were identified (Table 3). The information presented in Table 3 confirmed the previous finding that with increasing RCR, the interlamellar regions initially demonstrate a higher sensitivity to swelling. However, in the sample with the highest compression ratio (RCR = 6.05), this sensitivity was measurably reduced. This conclusion was also supported by the difference in the quantity of absorbed swelling agent: ≈12–15 wt % for the samples with an RCR of 1.29–2.48 compared to ≈10 wt % for PP_(6.05)_. Lastly, using data on the swelling-induced change of the thickness of amorphous layers (ΔLP, Table 3) and the initial thickness of these regions, the local strain in the amorphous regions was calculated (eq 8 and Table 3).

**Table 3:** Selected Structural Parameters of Undeformed and Compressed Polypropylene

Sample	Swelling-induced change of the thickness of amorphous layers (ΔLP) [nm]^a^	Local strain of amorphous phase (ε_a_) [-]^b^	Local stress of amorphous phase (σ_a_) [MPa]^c^	Elastic modulus of interlamellar amorphous phase [MPa]^d^
PP_(1)_	1.2	0.129	8.2	63.6 ± 0.1
PP_(1.29)_	1.4	0.133	4.4	33.1 ± 0.4
PP_(1.77)_	2.0	0.220	3.6	16.4 ± 0.1
PP_(1.84)_	2.0	0.217	4.1	18.9 ± 0.5
PP_(2.48)_	1.8	0.196	4.2	21.4 ± 0.9
PP_(2.93)_	1.1	0.120	4.5	37.5 ± 2.5
PP_(6.05)_	0.9	0.102	7.6	74.5 ± 1.1

a from SAXS.

b in accordance with eq 8.

c from the stress “buildup” experiment.

d in accordance with eq 6.

**Figure 10:**
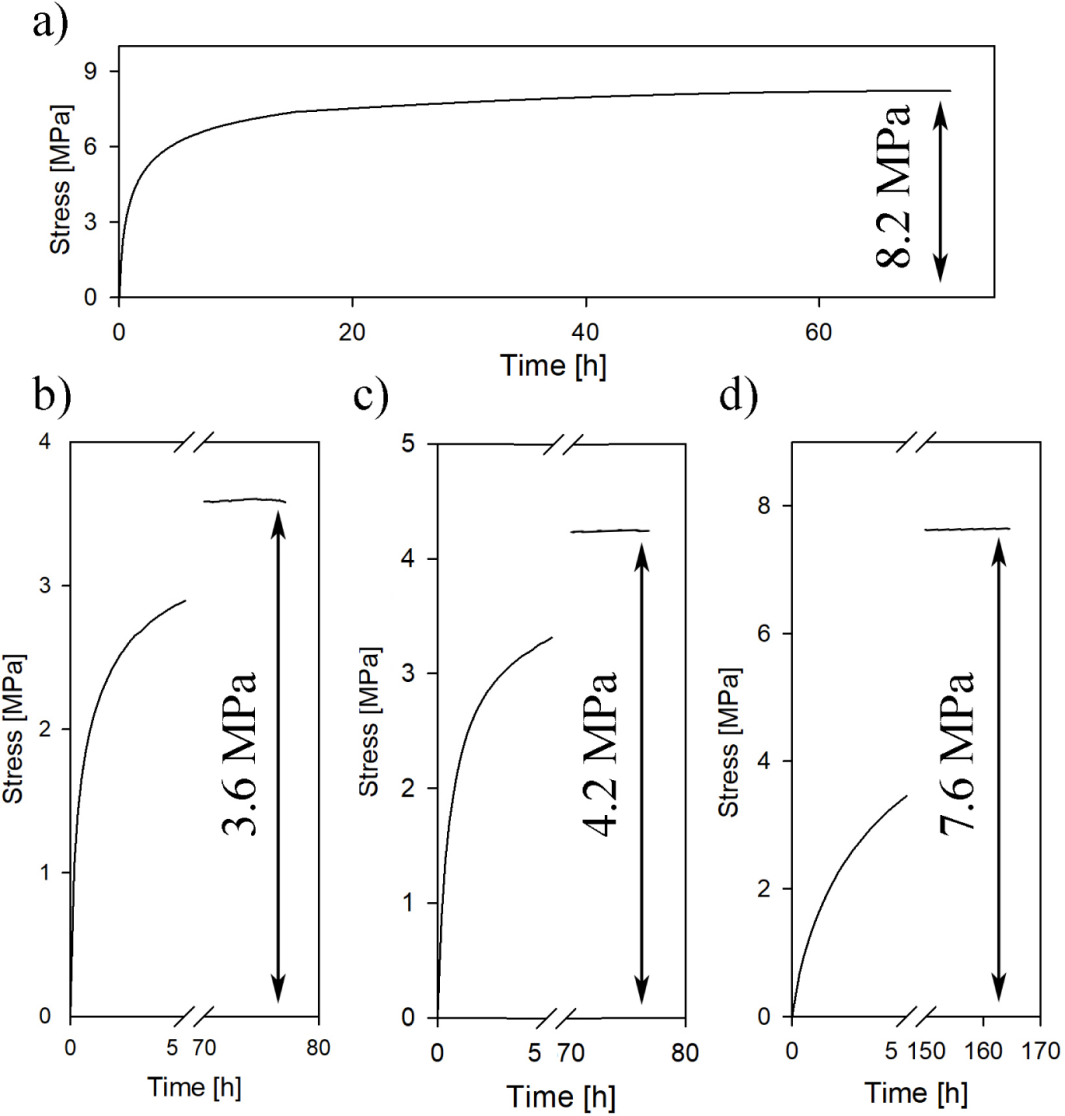
“Stress buildup” during the desorption of hexane for samples with the following values of RCR: a) 1, b) 1.77, c) 2.48, and d) 6.05.

The local stress in the amorphous regions of swollen materials was determined using the previously described method of stress “buildup” during the desorption of the swelling agent (see Subsection 3.1.2). Figures 10 and S9 show the results of stress “buildup” experiments conducted during hexane desorption for both undeformed and compressed materials. For the PP_(1)_ material (Figure 10a), hexane desorption was completed after 70 h, resulting in a stress buildup of 8.2 MPa. This stress level was slightly lower than the difference in yield stress observed between the reference polypropylene and the octane-swollen polypropylene discussed in Subsection 3.3 (10.1 MPa for PP_(25)_, Table 2). The discrepancy is attributed to the differences in the microstructures of the PP_(25)_ and PP_(1)_ samples, as discussed previously. Initially, the stress measured during hexane desorption showed a noticeable decrease for the PP_(1.29)_ and PP_(1.77)_ materials. However, with samples subjected to a higher compression ratio, the stress gradually increased, peaking at 7.6 MPa for the PP_(6.05)_ sample. The swelling-induced local stress in the amorphous layers (σ_a_) for all of the analyzed materials is summarized in Table 3.

**Figure 11:**
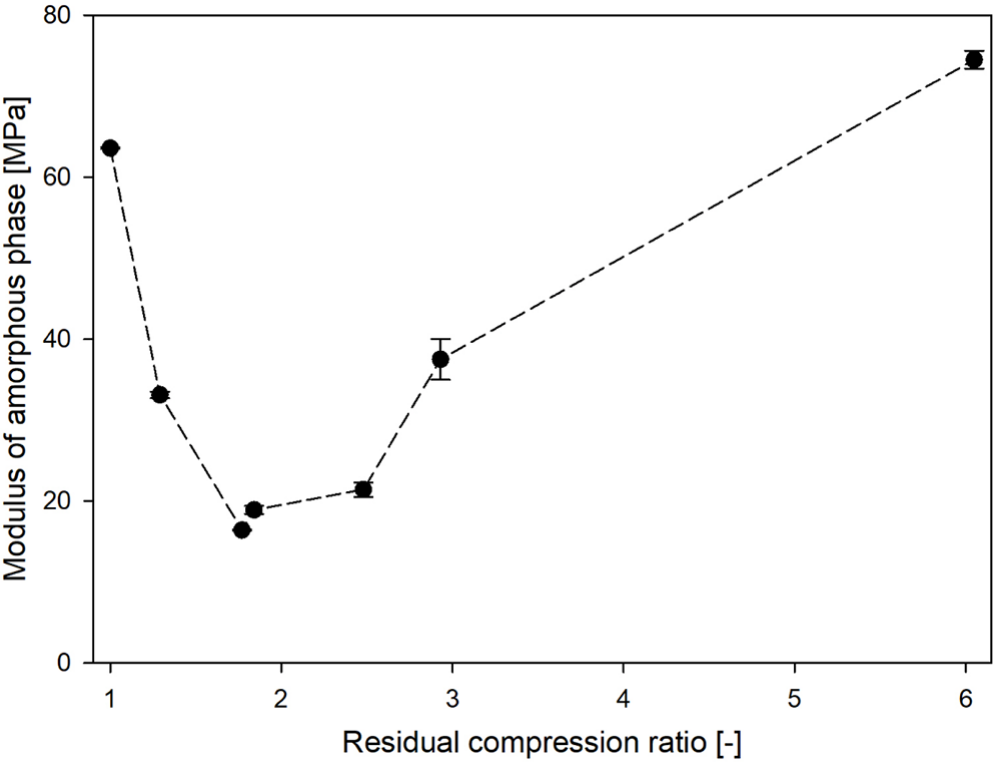
Elastic modulus of the interlamellar amorphous phase of the analyzed polypropylene as a function of the residual compression ratio. The dashed line is drawn only for eyes guidance.

The local strain/stress values were subsequently utilized to calculate the *E*_a_ values following the method outlined in eq 6 (Table 3). Furthermore, Figure 11 illustrates how *E*_a_ changes with varying RCR. A noticeable difference in the modulus was observed depending on the RCR. For the PP_(1)_ material, *E*_a_ was estimated at 63.6 MPa, which is measurably lower than the value obtained for the material discussed in Subsection 3.3 at ambient temperature (PP_(25)_, 80.8 MPa). It is worth mentioning that in our previous study, we noted a direct relationship between the *E*_a_ and crystal thickness for HDPE.^45^ Therefore, the observed discrepancy is mainly attributed to the thinner crystals in PP_(1)_ (5.1 nm) compared to those in PP_(25)_ (5.8 nm).

Surprisingly, the PP_(1.29)_ sample exhibited a significant decrease in the *E*_a_ value, nearly 2-fold, to 33.1 MPa. For sample PP_(1.77)_, this decrease in the modulus is even greater, nearly 4-fold, down to 16.4 MPa. An increase in stiffness of the amorphous phase was observed only in the PP_(1.84)_ and PP_(2.48)_ samples, where the modulus rose to 18.9 and 21.4 MPa, respectively. However, this value remained considerably below that of the uncompressed material. A major shift occurred when the sample was compressed to a RCR of 2.93 and 6.05. For the PP_(6.05)_ material, the *E*_a_ reached 74.5 MPa, which was ≈20% greater than the value for the nondeformed sample.

The initial decrease in the *E*_a_ value is primarily driven by the lamellae fragmentation process, which is most intense within the RCR range of 1–2.43 (Figure 6). The fragmentation of lamellar crystals into smaller segments/blocks helps to ease certain spatial restrictions imposed on the amorphous component. These spatial restrictions ordinarily arise from both the crystalline skeleton formed during solidification and the inherent microstructure of the amorphous regions, which includes a network of entanglements and tie molecules connecting adjacent lamellae. Furthermore, at this stage of deformation, the orientation of the molecular network along the *E*_a_ measurement direction remains low within both crystalline and amorphous components (Figure 5). Together, these factors influence the mobility of the amorphous phase, ultimately reducing its rigidity, which in turn leads to a 4-fold reduction in the *E*_a_ value.

**Figure 12:**
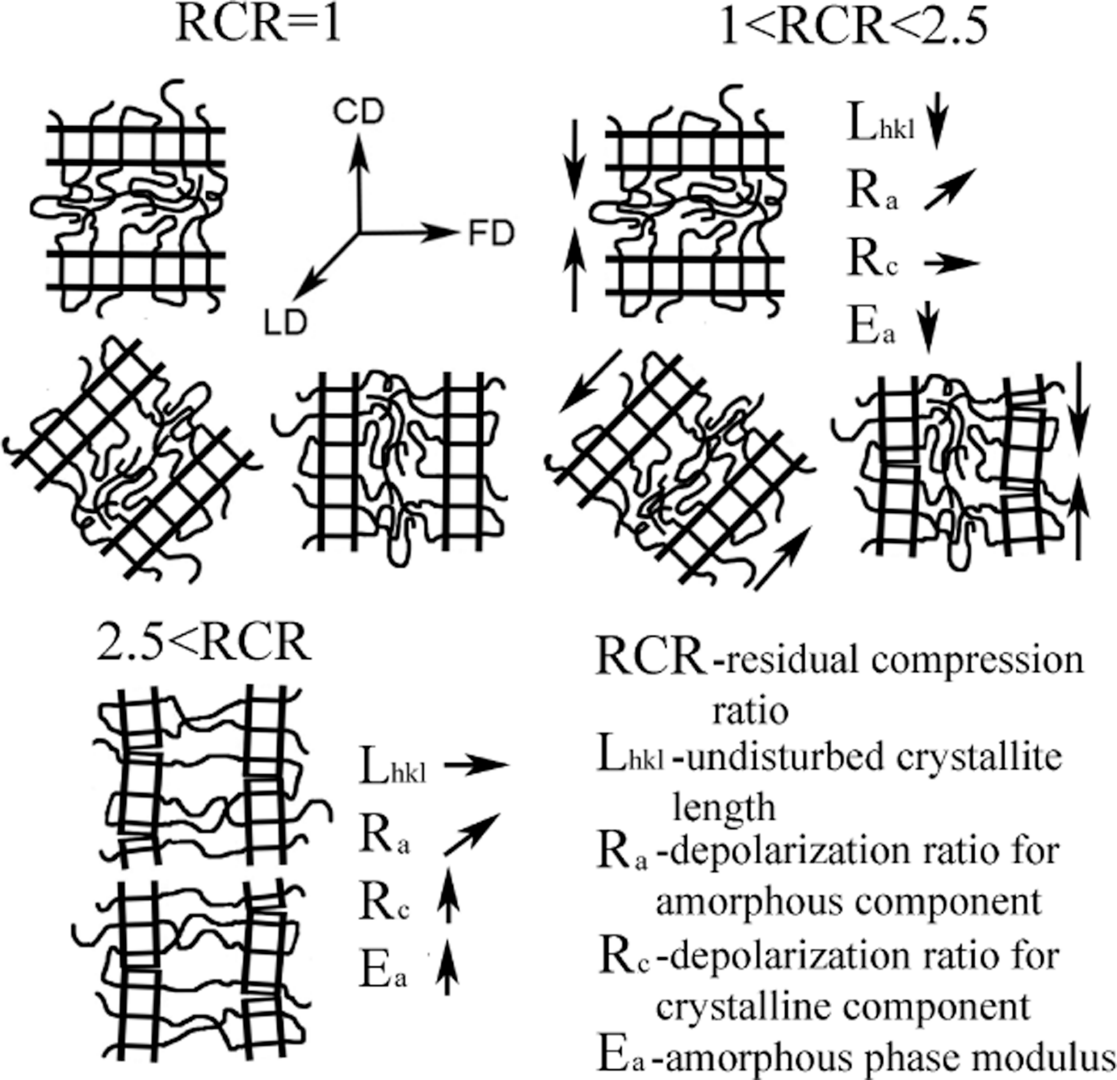
Schematic visualization of changes in the polypropylene microstructure during compression in a channel die.

At higher compression ratios (>2.43), the crystal fragmentation processes become less active (Figure 6). Simultaneously, there is a gradual increase in the molecular orientation along the *E*_a_ measurement direction within both the crystalline and amorphous regions (Figure 5). This increase in orientation is accompanied by a gradual stretching of the molecular network in the amorphous regions, ultimately introducing new geometrical restrictions on this component. Consequently, lateral contraction within the intercrystalline regions becomes notably restricted. As a result, the *E*_a_ value rises, as seen in PP_(2.93)_ and PP_(6.05)_, reaching a level nearly 20% higher than that of the reference/uncompressed sample in materials with the highest compression ratio. A schematic visualization of changes in the polypropylene microstructure during compression in a channel die is presented in Figure 12.

## 4 Conclusions

The decrease in the elastic modulus of PP’s amorphous regions with rising temperature, starting from ambient conditions, was clarified. The observed reduction of *E*_a_ results from a gradual increase in the mobility of chain fragments within the crystalline regions, triggered by the α relaxation process with rising temperature. This progressively diminishes the “stiffening” effect of lamellar crystals on the amorphous regions. On the other hand, lowering the temperature close to the glass transition point causes a significant increase in the *E*_a_ value, reaching nearly 70 MPa. Simultaneously, a deviation from the linear trend of *E*_a_ change with temperature (observed at higher temperatures) was noted.

The impact of both the undisturbed crystallite length and the orientation of the crystalline and amorphous components on *E*_a_ was also examined. These studies were carried out using PP samples compressed in a channel die with varying compression ratios, mainly to eliminate micronecking and cavitation processes. With increased compression ratios, two competing effects were observed that influence the *E*_a_ value. At lower compression ratios, the average dimension of lamellar crystals decreases, as the lamellae kink and fragment into smaller blocks in the absence of significant orientation of either the crystalline or amorphous components. These changes relieve some geometric constraints imposed on the amorphous phase by the crystalline framework formed during solidification, allowing the amorphous regions to contract in the lateral directions. Together, these factors increase mobility in the amorphous phase, reducing its stiffness and resulting in a significantly lower *E*_a_ value compared to the uncompressed material.

At moderate to high compression ratios, crystal fragmentation processes become less active. Concurrently, the orientation of chains in both crystalline and amorphous regions gradually accumulates along the direction of the *E*_a_ measurements. This alignment is accompanied by the stretching of the amorphous molecular network, generating new geometric constraints on the amorphous regions. Consequently, *E*_a_ increased, reaching a value nearly 20% higher than that of the reference sample in materials with the highest compression ratio.

## Supporting Information Available

Exemplary results of the deconvolution process of Raman spectra, WAXS profiles for reference/swollen polypropylene and corresponding X-ray diffraction spacings and crystallite lengths, representative compression curve, exemplary results of the deconvolution process of WAXS profiles, DSC and DMTA curves for isotropic polypropylene, SAXS profiles for reference and swollen samples as a function of RCR, and “stress buildup” curves during the desorption of hexane for samples with selected values of RCR (PDF)
