# Supplementary material for: Temperature and Deformation-Induced Changes in the Mechanical Properties of the Amorphous Regions of Semicrystalline Polypropylene
Source: J Phys Chem B. 2025 Jan 17;129(4):1426–38. doi: 10.1021/acs.jpcb.4c07863 (PMC11789131; doi:10.1021/acs.jpcb.4c07863)
Supplement: Supplementary file 1 — jp4c07863_si_001.pdf [file jp4c07863_si_001.pdf]

Temperature and Deformation Induced Changes in the Mechanical Properties of the Amorphous Regions of Semicrystalline Polypropylene

Małgorzata Polinska[1], Marcin Kozanecki[2], Artur Rozanski[1,*]

[1]Centre of Molecular and Macromolecular Studies, Polish Academy of Sciences,

Sienkiewicza 112, 90-363 Lodz, Poland

[2]Department of Molecular Physics, Faculty of Chemistry, Lodz University of Technology, Zeromskiego 116, Lodz, 90-924, Poland

[*]Corresponding author, e-mail: artur.rozanski@cbmm.lodz.pl

a)

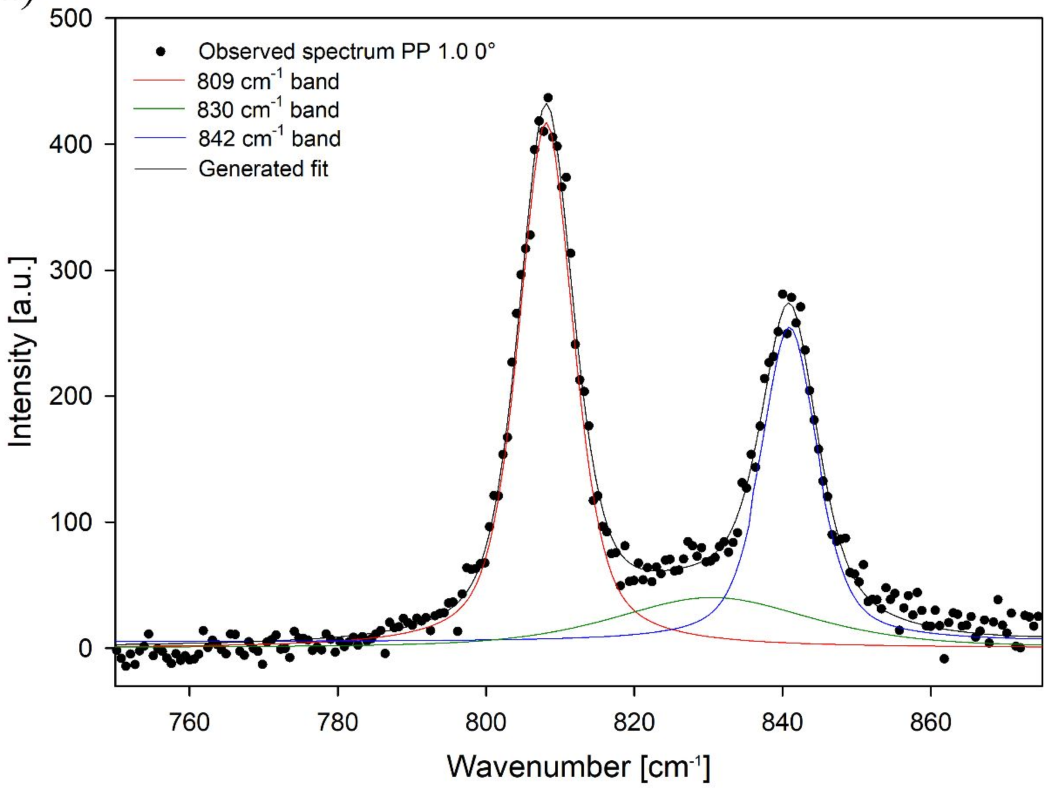

Observed spectrum PP 1.0 0°
809 cm-1 band
830 cm-1 band
842 cm-1 band
Generated fit
Intensity [a.u.]
Wavenumber [cm-1]
500
400
300
200
100
0
760
780
800
820
840
860

b)

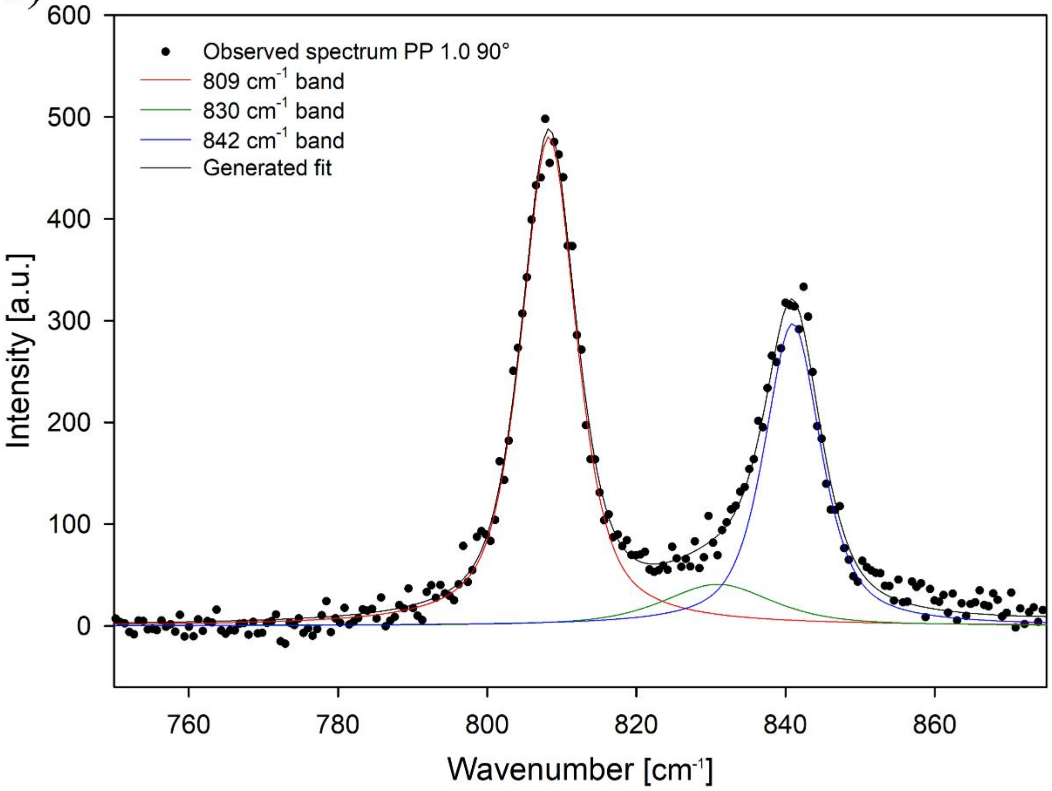

Observed spectrum PP 1.0 90°
809 cm-1 band
830 cm-1 band
842 cm-1 band
Generated fit
Intensity [a.u.]
Wavenumber [cm-1]
600
500
400
300
200
100
0
760
780
800
820
840
860

Fig. S1. The representative results of peak separation applied to the Raman spectra of the $PP_{(1)}$ sample with: a) 0° and b) 90° angles between the polarization of the incident light and the expected chain orientation.

a)

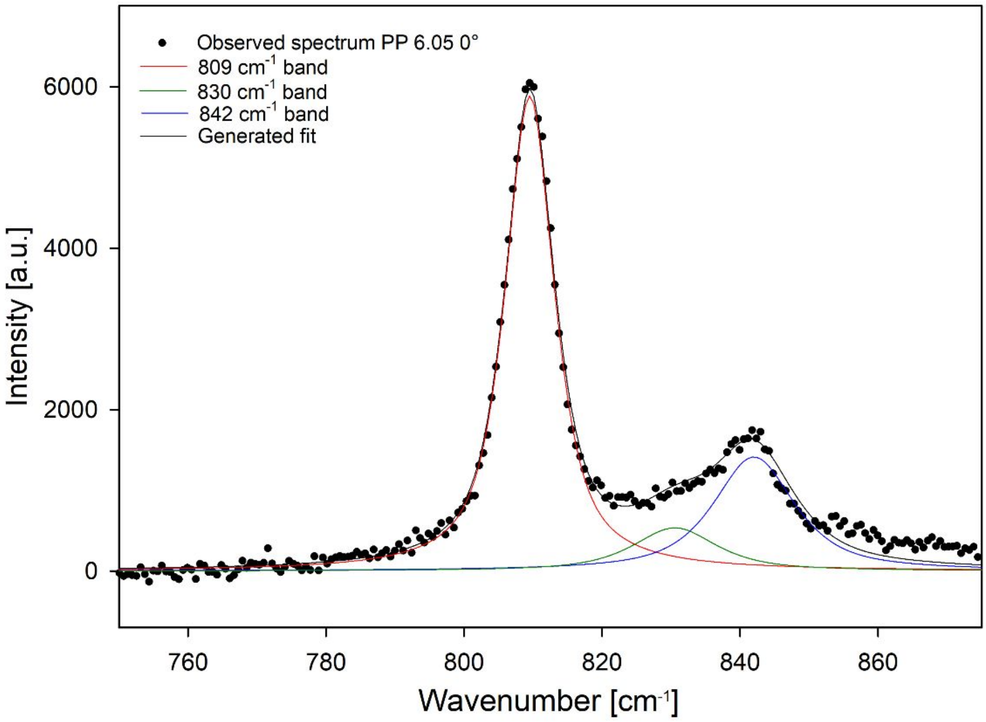

b)

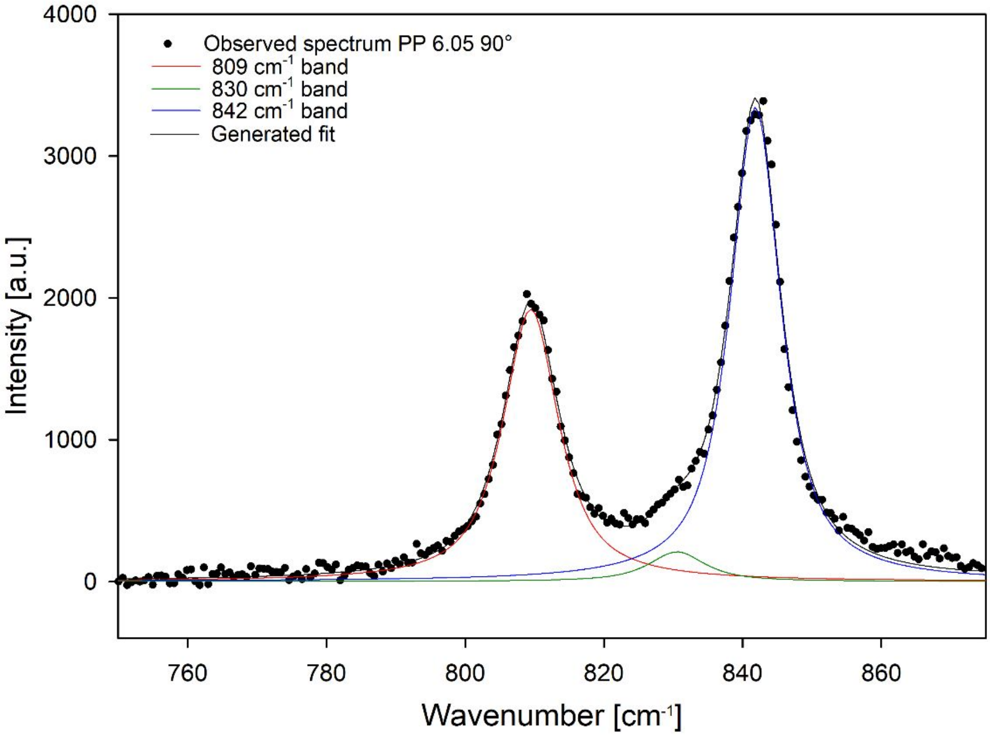

Fig. S2. The representative results of peak separation applied to the Raman spectra of the $PP_{(6.05)}$ sample with: a) 0° and b) 90° angles between the polarization of the incident light and the expected chain orientation.

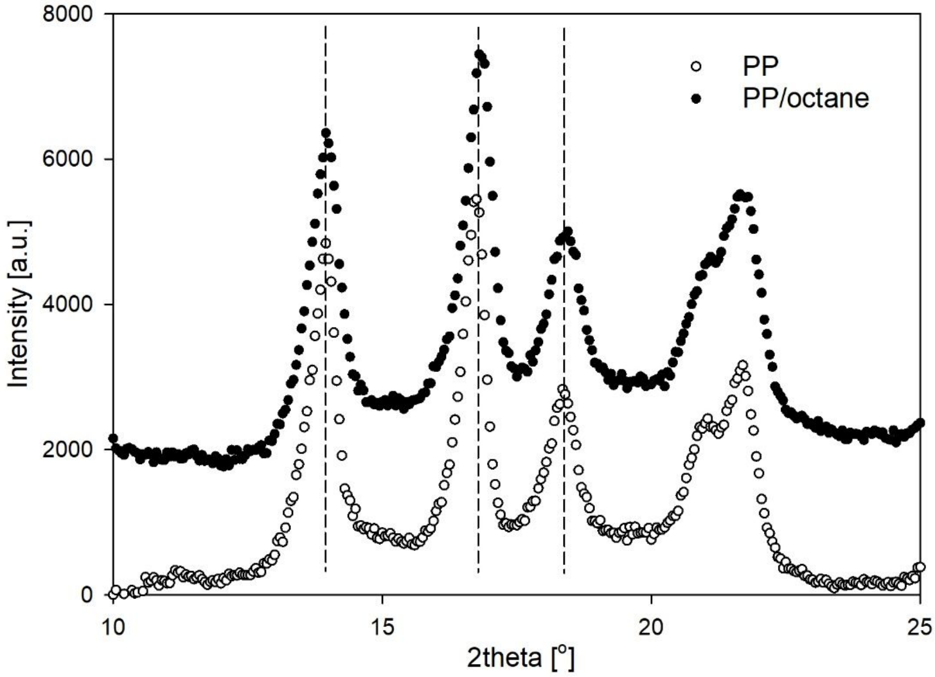

Fig. S3 WAXS profiles for reference and swollen polypropylene.

Tab. S1 X-ray diffraction spacings and crystallite lengths in the direction perpendicular to (hkl) planes

	d_{110} (nm)	d_{040} (nm)	d_{130} (nm)	L_{110} (nm)	L_{040} (nm)	L_{130} (nm)
PP	0.636	0.527	0.482	18.1	19.9	16.3
PP/octane	0.636	0.529	0.482	18.1	19.9	16.3

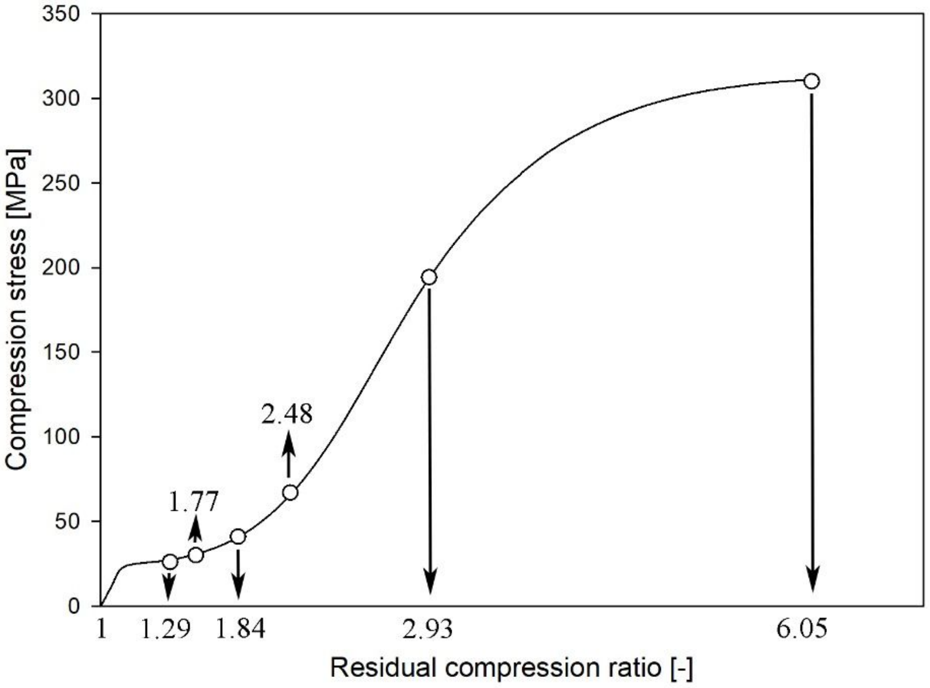

Fig. S4. Representative compression curve with marked RCR values analyzed in the work.

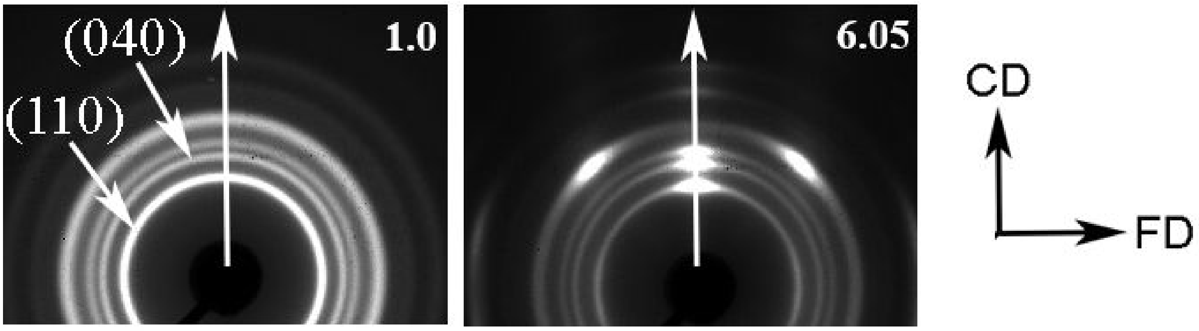

Fig. S5 2D WAXS patterns for samples with RCR of 1 and 6.05. The localization of the signal coming from (110) and (040) crystallographic planes. The arrow indicates the direction along which WAXS profiles were collected.

a)

b)

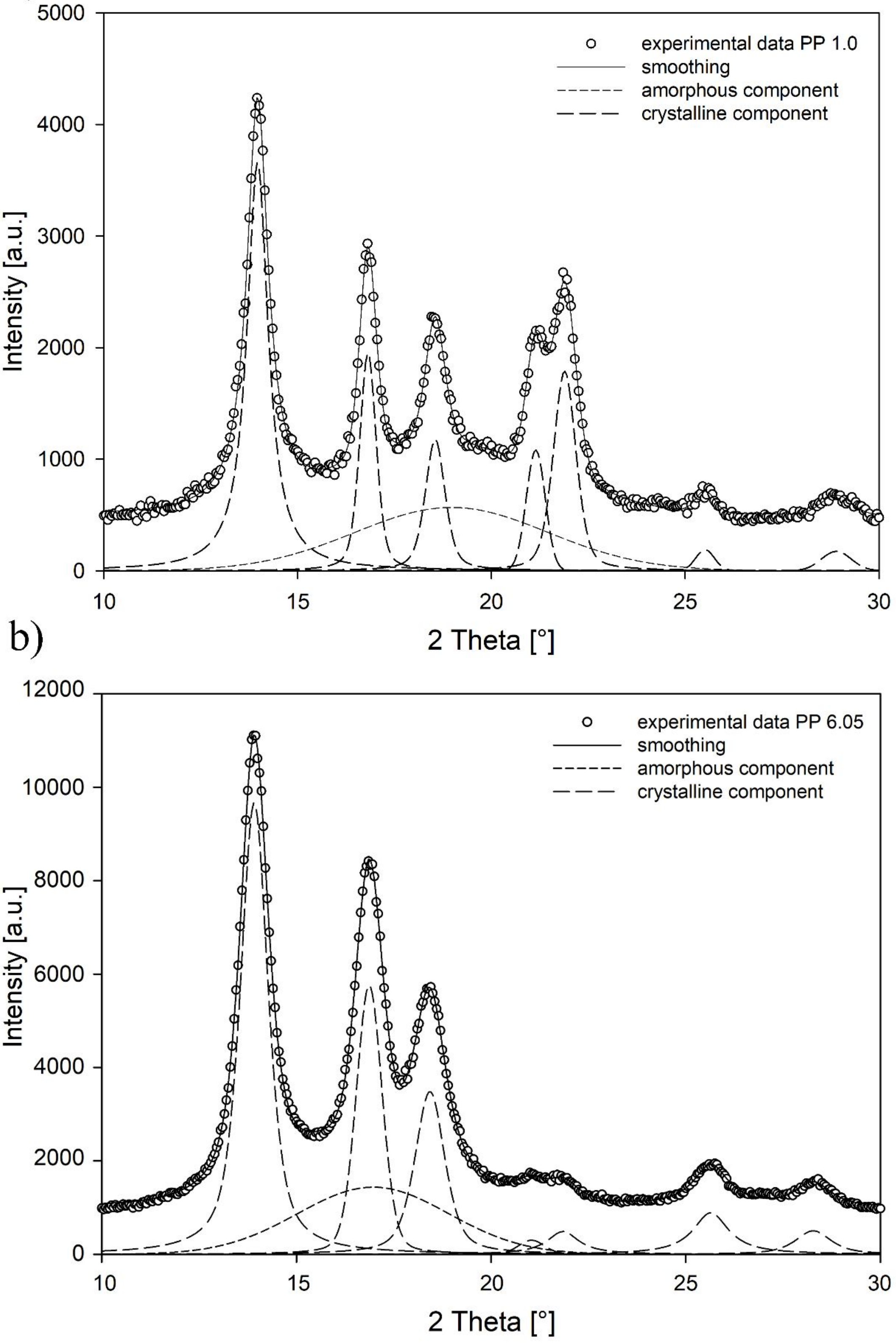

Fig. S6. The representative results of peak separation applied to the polypropylene with RCR values: a) 1, b) 6.05. The experimental data and smoothing curves were shifted along the vertical axis for better visualization.

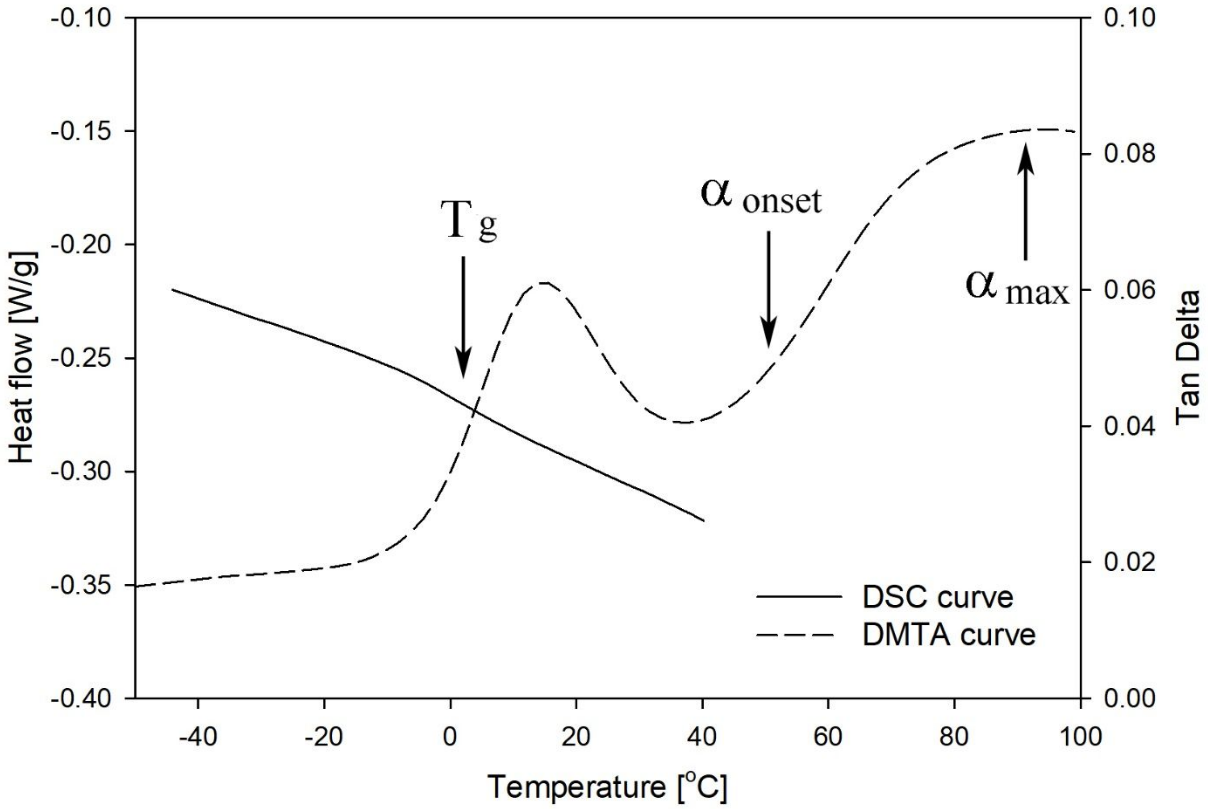

Fig. S7 Relations between heat flow/tan δ and temperature for analyzed polypropylene.

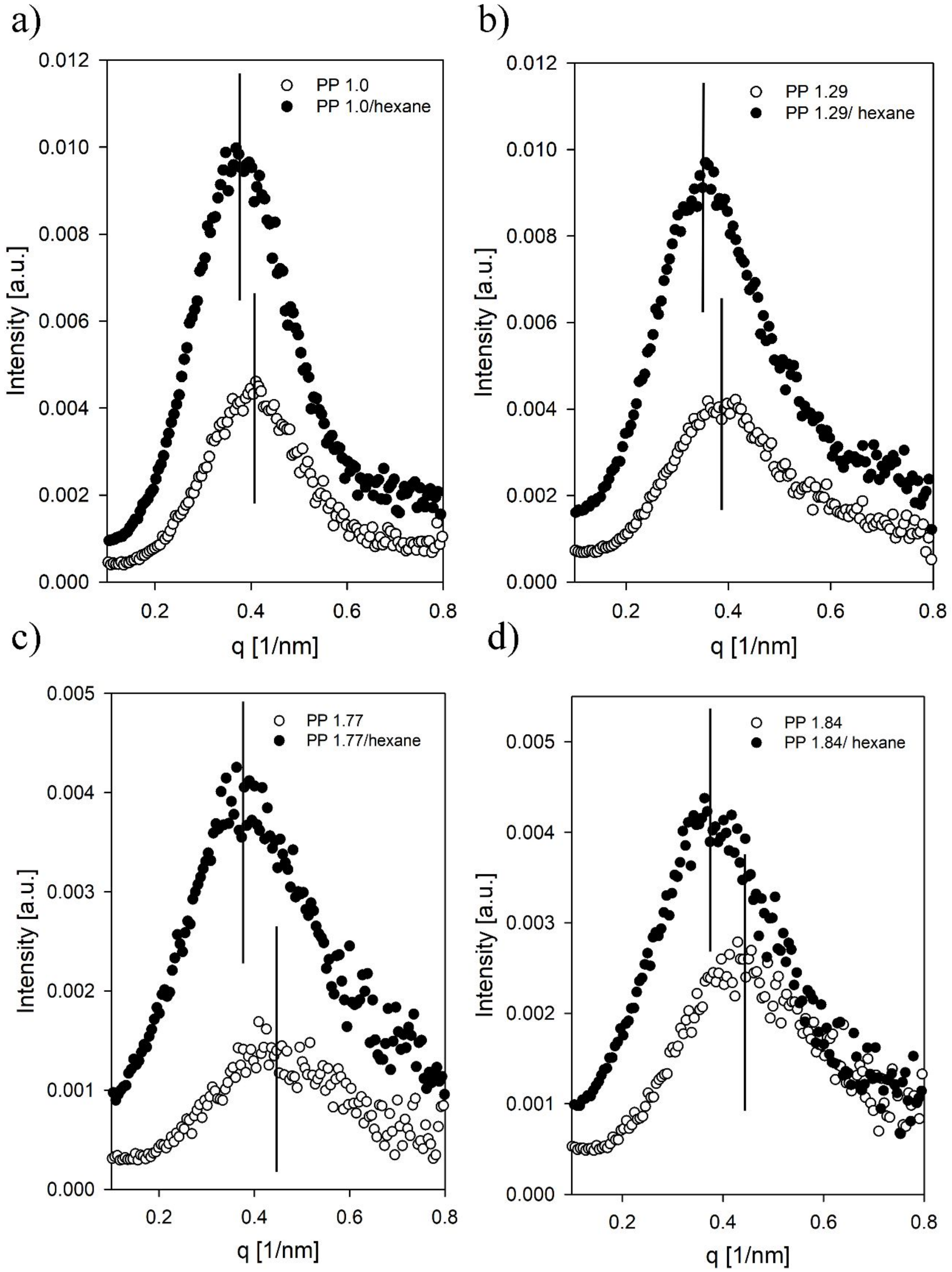

Fig. S8: SAXS profiles for reference (PP) and swollen (PP/hexane) samples as a function of RCR: a) 1, b) 1.29, c) 1.77, d) 1.84, e) 2.48, f) 2.93, g) 6.05.

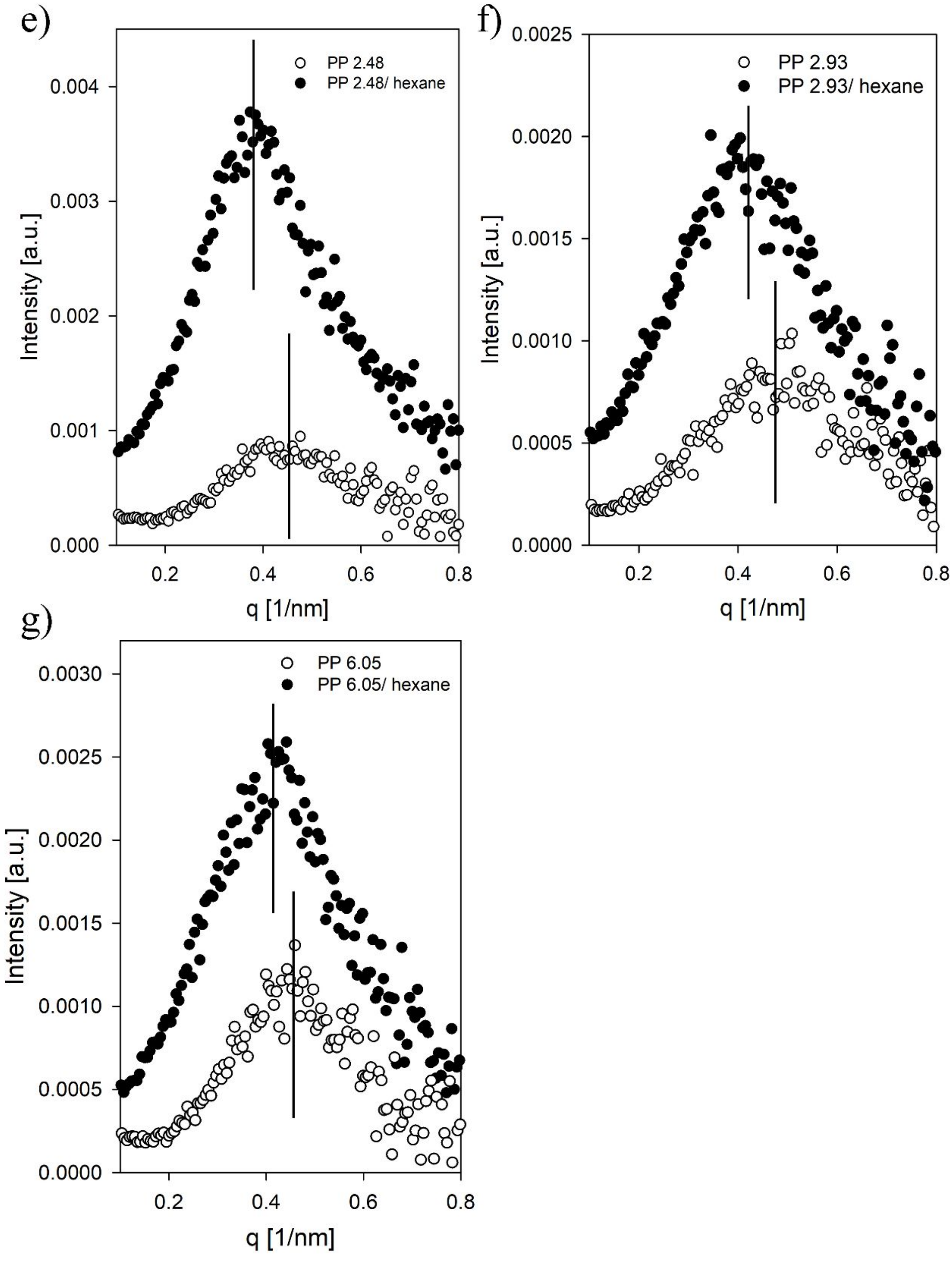

Fig. S8 (Continued from the previous page)

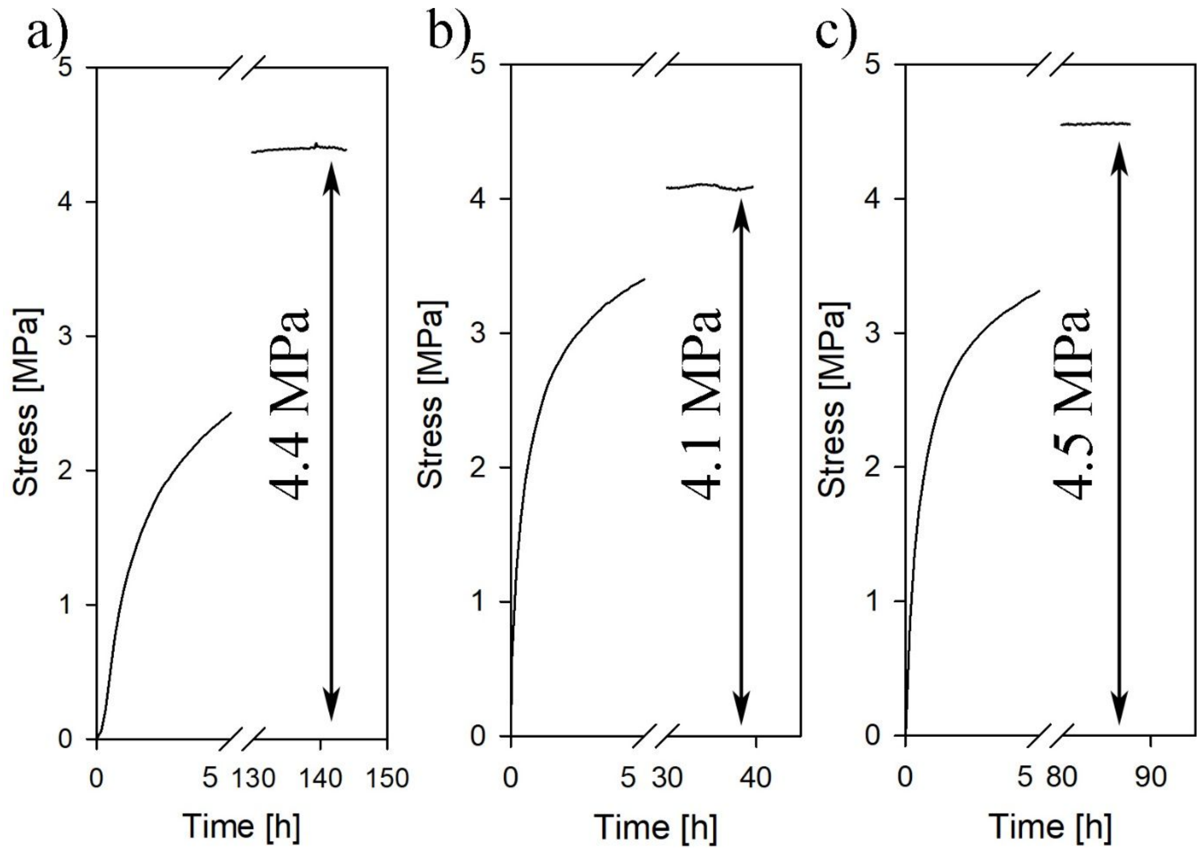

Fig. S9 The “stress build-up” during the desorption of hexane from samples with the following values of RCR: a) 1.29, b) 1.84, c) 2.93.
